# Context association in pyramidal neurons through local synaptic plasticity in apical dendrites

**DOI:** 10.3389/fnins.2023.1276706

**Published:** 2024-01-31

**Authors:** Maximilian Baronig, Robert Legenstein

**Affiliations:** Institute of Theoretical Computer Science, Graz University of Technology, Graz, Austria

**Keywords:** synaptic plasticity, dendrites, synaptic clustering, context-dependent computation, continual learning, unsupervised learning, top-down processing

## Abstract

The unique characteristics of neocortical pyramidal neurons are thought to be crucial for many aspects of information processing and learning in the brain. Experimental data suggests that their segregation into two distinct compartments, the basal dendrites close to the soma and the apical dendrites branching out from the thick apical dendritic tuft, plays an essential role in cortical organization. A recent hypothesis states that layer 5 pyramidal cells associate top-down contextual information arriving at their apical tuft with features of the sensory input that predominantly arrives at their basal dendrites. It has however remained unclear whether such context association could be established by synaptic plasticity processes. In this work, we formalize the objective of such context association learning through a mathematical loss function and derive a plasticity rule for apical synapses that optimizes this loss. The resulting plasticity rule utilizes information that is available either locally at the synapse, through branch-local NMDA spikes, or through global Ca^2+^events, both of which have been observed experimentally in layer 5 pyramidal cells. We show in computer simulations that the plasticity rule enables pyramidal cells to associate top-down contextual input patterns with high somatic activity. Furthermore, it enables networks of pyramidal neuron models to perform context-dependent tasks and enables continual learning by allocating new dendritic branches to novel contexts.

## 1 Introduction

The integration of bottom-up sensory input with top-down contextual information is a core computational principle of the neocortical microcircuit (Gilbert and Li, 2013; Schuman et al., 2021). In this way, sensory processing in cortex is enriched with behavioral context such as attention, expectations, and task information. Pyramidal neurons in neocortical layers 2/3 and 5 are assumed to play a pivotal role in this computation since the morphological structure of these cells is well-aligned to integrate the two information streams. Feed-forward input from the thalamus or from areas located lower in the cortical hierarchy are relayed via layer 4 to the basal dendrites of the pyramidal cells. On the other hand, top-down input from higher cortical areas targets mainly neocortical layer 1, where it reaches the apical tuft of these cells (Felleman and Van Essen, 1991; Cauller et al., 1998; Shipp, 2007). The dendrites of the apical tuft are electrotonically segregated from the basal dendrites, allowing for an independent integration of these two signals (Schuman et al., 2021). Their integration within the cell is based on a repertoire of nonlinear dendritic processes. In particular, the dendrites of layer 5 pyramidal neurons (L5Ps) exhibit several types of dendritic spikes including NMDA spikes (regenerative processes that depend on N-methyl-D-aspartate receptors) and Calcium (Ca^2+^) spikes (Spruston, 2008; Major et al., 2013; Stuyt et al., 2022).

This unique architecture has stimulated a number of theories about the organization of cortical computation and the role of top-down projections (Rao and Ballard, 1999; Bar, 2003; Lee and Mumford, 2003; Larkum, 2013; Keller and Mrsic-Flogel, 2018; Acharya et al., 2022). These proposals include computations based on attention (Bar, 2003), predictions (Keller and Mrsic-Flogel, 2018), associations (Larkum, 2013), and further extend to the role of this integration in conscious processing (Aru et al., 2020). In particular, Larkum (2013) proposed that L5Ps detect the coincidence of bottom-up activity and top-down activations indicating context information and associate the activity patterns in these two information streams. However, it is not well-understood how synaptic plasticity at the apical dendrites could support such associations. Experimental evidence points to an organization of learning where apical inputs from different contexts are segregated (Cichon and Gan, 2015), presumably due to the spatial clustering of functionally related inputs on dendritic branches (Kleindienst et al., 2011; Fu et al., 2012; Takahashi et al., 2012; Wilson et al., 2016; Kastellakis and Poirazi, 2019). In this article, we ask to what extent cell-internal signals can give rise to synaptic plasticity rules at apical synapses that support the formation of context-associations in pyramidal cells. To investigate this question, we have chosen a top-down approach. Starting from three main hypotheses on the properties and role of apical plasticity, we postulate a loss function that captures the goals of the learning process. These hypotheses are that the main objectives of plasticity are (1) to associate contextual input patterns that co-occur with strong basal activity to the neuron, (2) to cluster co-active synapses at the apical tuft, and (3) to segregate apical activity induced by different contextual input patterns. We then derive in a simplified pyramidal cell model a plasticity rule for apical synapses that approximates gradient descent on this loss function. We show that the resulting synaptic plasticity rule relies on the main established cell-internal signaling mechanisms and variables locally available to the synapse. We refer to this learning rule as the context-association learning (CAL) rule. We further investigate the functional properties of the CAL rule in networks of pyramidal neurons. The results of computer simulations confirm that the CAL rule provides the basis for the association of top-down context information with relevant bottom up input features in pyramidal neurons. When applied in a network in combination with unsupervised adaptation of basal synaptic efficacies, this allows the network to learn a multi-task classification problem. Furthermore, we show that the network can also learn to solve this problem in a continual manner, where performance on previously learned tasks is maintained after new tasks have been acquired by the network. This continual learning capability is possible because novel context patterns are associated to the neurons on newly recruited dendritic branches while previously used branches are protected from plasitcity.

Our results suggest that cell-internal signaling is a key component for synaptic plasticity that controls the integration of bottom up input with top-down contextual information, enabling context-dependent learning at the network level.

## 2 Results

### 2.1 A simplified pyramidal cell model for context-dependent computation

**Figure 1:**
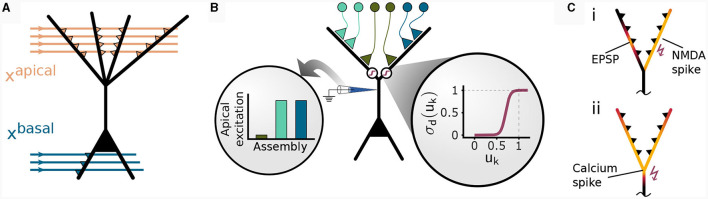
Non-linear input integration in our pyramidal neuron model. **(A)** Schematic illustration. The basal integration site receives sensory feed-forward input *x*^basal^ and integrates it as a weighted sum of inputs in a single compartment. The apical compartment receives context input vector *x*^apical^ and integrates it in each of its *K* branches. **(B)** Clustered input results in higher apical excitation *e*^a^ than input scattered on different branches (left circular inset) due to apical branch non-linearity σ_d_(*u_k_*) (right circular inset). **(C)** Local and global dendritic events in our model. (i) Local synaptic input induces excitatory post-synaptic potentials (EPSPs). NMDA-spikes $s_{k}^{\text{NMDA}}$signal branch-wide information, enabling clustering via potentiation of co-active synapses within the same branch (Brandalise et al., 2022). (ii) Calcium spikes are regenerative dendritic events spreading across the apical dendritic tree. This mechanism can account for the signaling of information about the total activation of the apical tuft.

We considered an abstract model for a cortical pyramidal neuron that consists of two sites of dendritic integration: a basal site and an apical site, see Figure 1A. The *apical* site receives a binary context input vector $x^{\text{apical}}\in {\left\{0,1\right\}}^{n^{\text{apical}}}$, where *n*apical is the number of synapses on each branch, at its *K* dendritic branches. Each branch *k* ∈ {1, …, *K*} computes a local branch membrane potential *u_k_* as


(1) $$u_{k}=\sum_{i}x_{i}^{\text{apical}}w_{ki},$$

where *i* denotes the synapse index and $w_{k}\in {\mathbb{R}}^{n^{\text{apical}}}$ the branch-specific synaptic weight vector with each weight *w*_*ki*_ constrained between 0 and a maximal weight *w*_max_.

From this local branch potential *u_k_*, the probability of a dendritic NMDA spike is given for each branch by a generalized sigmoid function (see Figure 1B, inset and *Methods*)


(2) $$p_{k}=p(s_{k}^{\text{NMDA}}=1|u_{k})=\sigma_{\text{d}}(u_{k}).$$

A similar non-linear transfer function modeling active dendritic properties has been used in Ujfalussy and Makara (2020), where the authors found that a strongly supra-linear transfer function is required for synaptic clustering. An NMDA spike at branch *k* is then elicited with probability *p*_*k*_. Due to the non-linearity σ_d_, depolarization due to synapses within the same dendritic branch results in a higher apical excitation than the same input distributed between multiple branches (Figure 1B). More formally, we denote an NMDA spike in branch *k* by $s_{k}^{\text{NMDA}}$ ∈ {0, 1} which is drawn from a Bernoulli distribution with mean *p*_*k*_: $s_{k}^{\text{NMDA}}$~B(*p*_*k*_).

In the *basal* compartment, synaptic weights *v_i_* are used to transform basal input vector *x*^basal^ ∈ ${\mathbb{R}}^{n^{\text{basal}}}$ into a basal membrane potential *u*^b^ ∈ ℝ according to


(3) $$u^{b}=\sum_{i}x_{i}^{\text{basal}}v_{i},$$

with synapse index *i*.

A calcium spike is triggered when sufficient apical activation coincides with sufficient basal activity (Larkum et al., 1999; Larkum, 2013). In our model, the apical activation is determined by the number of NMDA spikes generated over all *K* branches


(4) $$u^{a}=\sum_{k\in K}s_{k}^{\text{NMDA}}.$$

Formally, the model elicits a Ca^2+^spike when both the basal voltage *u*^b^ and the apical activation *u*^a^ are above their respective thresholds θ^b^ and *n*^Ca^


(5) $$S^{\text{Ca}}=\{\begin{array}{ll} 1, & \text{if }u^{\text{b}}\geq \theta^{\text{b}}\text{ and }u^{\text{a}}\geq \;n^{\text{Ca}}\\ 0 & \text{otherwise.} \end{array}$$

The amplitude of this calcium spike is fixed and independent from the number of dendritic NMDA spikes as long as the threshold *n*^Ca^ is reached. Hence, our model features different signals that are considered to act on different spatial scales within the neuron, see Figure 1C: Synapse-local synaptic input, branch-wide NMDA spikes, and tuft-wide Ca^2+^-spikes. The CAL rule described below combines these sources of information for synaptic updates.

The neuron output rate *r* is determined by the basal voltage, elevated by a scaled contribution of calcium spike *S*^Ca^ via


(6) $$r=u^{\text{b}}+\alpha S^{\text{Ca}},$$

with strength coefficient α. In our network simulations in Sections 2.5 and 2.6, we used a K-winner-take-all architecture which mimics the impact of lateral inhibition, transforming *u*^b^ before output rate *r* is calculated (see *Methods*).

For the analysis of the learning properties of our model, we would like a quantity that tells us how well the activation of the apical compartment supports the generation of Ca^2+^spikes. As such quantity, we used the expectation of generating a calcium spike, given that basal activity is above threshold. We term this quantity the apical excitation *e*^a^. It is formally defined as


(7) $$e^{\text{a}}=\;E[\Theta (u^{\text{a}}-n^{\text{Ca}})],$$

where Θ denotes the Heaviside step function Θ(*s*) = 1 if *s*≥0 and 0 otherwise. For given NMDA spike probabilities at the branches, this quantitiy can be computed analytically, see *Methods*.

### 2.2 A learning rule for context association

Experimental findings suggest that feed-forward bottom-up sensory stimuli target preferentially the basal dendrites of neocortical pyramidal neurons, whereas top-down contextual input may target the apical dendritic tuft (Gilbert and Li, 2013; Phillips, 2017; Schuman et al., 2021). It has been proposed that the association between these two distinct inputs through plasticity mechanisms that depend on the simultaneous basal and apical activity is a central computational primitive in these cells (Larkum, 2013). Back-propagation of somatic activity to the apical dendrites appears to be a biological implementation of this coincidence-based mechanism (Shai et al., 2015). There are many ways how such association could be implemented through synaptic plasticity mechanisms. Experimental evidence points to an organization of learning where apical inputs from different contexts are segregated (Cichon and Gan, 2015), presumably due to the spatial clustering of functionally related inputs on dendritic branches (Kastellakis and Poirazi, 2019; Limbacher and Legenstein, 2020). Given these findings, our aim was to derive a normative plasticity model that organizes synaptic inputs on apical dendrites with the following hypothetical aims:

**Objective 1 (association):** contextual input patterns that frequently co-occur with strong basal activity should induce strong apical activation.
**Objective 2 (clustering):** co-active synapses at the apical tuft should cluster on dendritic branches.
**Objective 3 (dissociaton):** contextual input patterns that rarely co-occur with strong basal activity should lead to weak apical activation.

Objective 3 ensures that not all contextual patterns are associated, which would lead to unspecific apical activation. We denote this learning principle as Context Association Learning (CAL) and the resulting plasticity rule as the CAL rule.

To investigate whether a plausible plasticity rule can fulfill these requirements, we formulated the above objectives in a mathematical manner through a loss function ${ℒ}_{\text{CAL}}$. This enabled us to derive a rule that performs gradient descent on this loss, i.e., a rule that updates apical synaptic weights such that the loss ${ℒ}_{\text{CAL}}$ is decreased, and therefore fulfills the requirement in a mathematically rigorous manner. The proposed loss function ${ℒ}_{\text{CAL}}$ is given by


(8) $${ℒ}_{\text{CAL}}=u^{\text{BP}}A+\lambda u^{\text{BP}}C+\kappa (1-\;u^{\text{BP}})D.$$

Here, *u*^BP^ ∈ {0, 1} denotes the backpropagated activity that is 1 if the basal activation *u*^b^ is above a threshold *θ*^b^ and 0 otherwise. The loss components A, C, and D implement the association-, clustering-, and dissociation-objectives described above respectively, where the parameters λ>0 and κ>0 define the relative contributions of these components. The association loss A and the dissociation loss D counteract each other and *u*^BP^ acts as gating variable selecting which of the two loss terms to minimize. A encourages increased apical activation (when *u*^BP^ is high) and D encourages decreased apical activity (when *u*^BP^ is low). In addition, when a context pattern is associated, the clustering loss C encourages a clustering of this input pattern. In particular, the association loss A is given by


(9) $$A=\text{max}\left(n^{\text{Ca}}-\sum_{k}s_{k}^{\text{NMDA}},0\right).$$

This term evaluates to 0 if at least *n*^Ca^ dendritic branches elicit an NMDA spike, the threshold for an apical Ca^2+^spike and hence penalizes insufficient NMDA spiking. In the following simulations, we have chosen *n*^Ca^ = 1 for simplicity if not noted otherwise (we also conducted experiments with *n*^Ca^ = 2, see Section 2.3, and *n*^Ca^ = 3, see Supplementary material, Section S3).

The clustering loss C is given by


(10) $$C=\sum_{k}\text{Var}(s_{k}^{\text{NMDA}}).$$

This term penalizes NMDA spike probabilities that lead to high variance of NMDA spiking. Since these NMDA spikes are sampled from a Bernoulli distribution, the variance is low if the spiking probability is either close to 0 or close to 1. Minimizing this variance induces distribution of firing activity across few strongly activated instead of multiple weakly activated branches. The parameter λ in Equation (8) controls the strength of this clustering objective, introducing a form of competition between branches (see also Supplementary material Section S1).

For the dissociation loss D we have chosen


(11) $$D=\sum_{k}\sigma_{\text{d}}(u_{k}).$$

**Figure 2:**
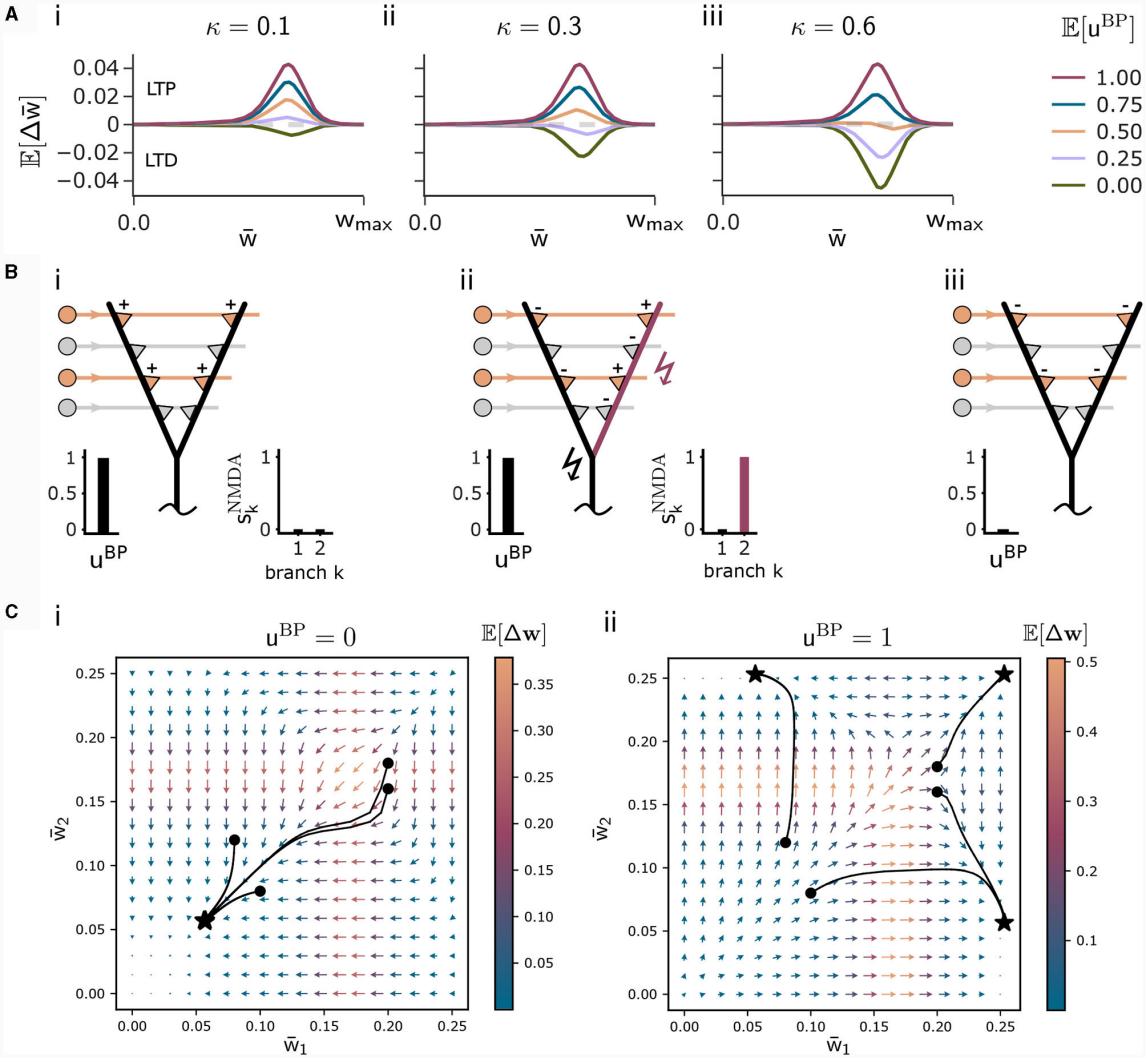
Illustration of the CAL rule. **(A)** Back-propagating activity *u*^BP^ switches between LTP (from loss A) and LTD (from loss D), balanced by κ. Shown are expected weight updates 𝔼[Δ*w*_*kj*_] of active synapses for different average levels of *u*^BP^ in a model with a single dendritic branch *k*, where all synaptic weights have the same value $w_{ki}=\bar{w}$. The dependence on the mean synaptic weight $\bar{w}$ arises from the dependence on the branch potential. The balance between potentiation and depression is defined by the parameter κ ((i) κ = 0.1, (ii) κ = 0.3, (iii) κ = 0.6). **(B)** Schematic illustration of weight updates with CAL. (i) Strong back-propagating activity *u*^BP^ = 1 in absence of a calcium spike (*S*^Ca^ = 0) results in LTP of depolarized synapses on all branches, due to the minimization of association loss A. (ii) Strong back-propagating activity *u*^BP^ = 1 coinciding with a calcium spike (*S*^Ca^ = 1, here *n*^Ca^ = 1), results in LTP of depolarized synapses on spiking branches and LTD in depolarized synapses on non-spiking branches, due to minimization of clustering loss C. **(iii)** Weak *u*^BP^ = 0 results in dissociation, due to dissociation loss D. **(C)** Phase plane analysis of weight update dynamics undergoing CAL. To project the high-dimensional weight updates on a 2D plane, we assigned all weights *w*_*kj*_ of activated synapses (*x*_*j*_ = 1) on branch *k* the same value ${\bar{w}}_{k}$. The x and y axes show these synaptic weights ${\bar{w}}_{1}$ and ${\bar{w}}_{2}$ respectively for a neuron model with two branches. Arrow color is proportional to the expected weight update $𝔼\left[\Delta {\bar{w}}_{j}\right]$, whereas the arrow length is proportional to its logarithm for visibility (see *Methods* for details). We show four trajectories of different initializations, where the dot refers to the initial point and the star to the final weight configuration for (i) *u*^BP^ = 0 and (ii) *u*^BP^ = 1.

This loss is high for high NMDA spike probabilities. For *u*^BP^ = 0, this loss governs the loss function (8) encouraging the plasticity rule to not associate context input patterns. The parameter κ in Equation (8) determines the relative influence of the association and the dissociation term. For small κ, patterns are likely associated even for infrequent backpropagating activity *u*^BP^. The higher κ, the more backpropagating activity is necessary to associate a pattern. The influence of this parameter on the weight change is illustrated in Figure 2A.

The plasticity rule for apical synaptic weights *w*_*kj*_ is then obtained by performing weight updates in the negative direction of the gradient of the loss function:


(12) $$\Delta w_{kj}=-\eta (w_{kj})\;\frac{\partial }{\partial w_{kj}}{ℒ}_{\text{CAL}},$$

where η(*w*_*kj*_) is a weight-dependent learning rate (see *Methods*). We derive in *Methods* the synaptic efficacy updates from this loss function, the CAL rule. The update for the *j*-th synaptic weight at branch *k* is given by


(13) $$\begin{array}{l} \Delta w_{kj}=\eta (w_{kj})\;[u^{\text{BP}}\underset{\text{from }A}{\underset{︸}{x_{j}f(u_{k})\left(1-S^{\text{Ca}}\right)}}\\ \;+\lambda \;u^{\text{BP}}\underset{\text{from }C}{\underset{︸}{x_{j}g(u_{k})\left(2s_{k}^{\text{NMDA}}-1\right)}}\\ \;-\kappa \;\left(1-u^{\text{BP}}\right)\underset{\text{from }D}{\underset{︸}{x_{j}g(u_{k})}}\\ \;-\lambda_{\text{reg}}\;u^{\text{BP}}h_{j}(u_{k},w_{k})], \end{array}$$

where *f*(*u*_*k*_) and *g*(*u*_*k*_) are non-negative functions that depend only on the local branch potential. Here, we added to the gradient a regularization term *h*_*j*_(*u*_*k*_, **w**_*k*_) which reduces weights of inactive synapses and scales synaptic efficacies to normalize the weights at each dendritic branch (see *Methods*). After applying the update, weights were clipped between 0 and a maximum weight *w*_max_.

We briefly discuss the biological interpretation of this rule. First, in the case of strong basal activity (*u*^BP^ = 1), the first and second line, that originate from minimization of the association loss A and clustering loss C, dominate. The first line of Equation (13) increases weights—and thus the expected number of NMDA spikes—until the Ca^2+^spike initiation threshold *n*^Ca^ is reached and a Ca^2+^spike appears (Figure 2Bi). Hence, the Ca^2+^spike determines the plasticity. When a Ca^2+^spike is present, the second line in the update rule (13) becomes most relevant. It depends on the local branch potential and local NMDA spikes. In particular, active synapses at branches with a local NMDA spike are potentiated while others are depressed. On average, the term (2$s_{k}^{\text{NMDA}}$−1) strengthens synapses at branches that are likely to produce NMDA spikes while it weakens those on branches which are unlikely to spike. This favors synaptic clusters as stronger clusters are reinforced. In effect, activity is concentrated on few branches (Figure 2Bii), where the number of active branches is determined by the threshold for Ca^2+^spike initiation *n*^Ca^.

Finally, in the case of weak basal activity (*u*^BP^ = 0), the third line of Equation (13) dominates, leading to depression of active synapses (Figure 2Biii). This term originates from minimization of the dissociation loss D.

To summarize, we found that the objectives 1–3 can be achieved by a synaptic plasticity rule that depends on (a) pre-synaptic activity, (b) the local branch potential, (c) NMDA spikes, and two more neuron-global signals, that is, (d) somatic activity backpropagating to the apical tuft, and (e) Ca^2+^ events. This result indicates that branch-wide signaling through NMDA spikes and tuft-wide signaling through Ca^2+^ spikes (Figure 1C) are sufficient to achieve the learning objectives.

To better understand the dynamics of this learning rule, we peformed a phase plane analysis for a neuron with two dendritic branches, see Figure 2C. In the case of no backpropagating activity *u*^BP^ = 0 (panel Ci), all weights of active synapses (synapses *j* where *x*_*j*_ = 1) converge to a low value, where the NMDA-spike probability of both branches is negligibly small. If *u*^BP^ = 1 however (panel Cii), the initial weights determine whether either active synapses of branch 1, branch 2, or both branches converge to strong efficacies. This analysis demonstrates that the learning rule instantiates a type of competition between branches to become active if *u*^BP^ = 1. The clustering loss C is essential for this competition, as the competition is diminished without it (see Supplementary material Section S1).

### 2.3 Pattern association with the context association learning rule

The primary objective of the CAL rule is the association of activity patterns presented to the apical dendritic branches with basal activity, such that apical activity that coincides with basal activity leads to strong apical activation. The objectives defined above further encourage that this association is performed in a clustered manner, i.e., the rule should preferentially lead to the activation of few branches for a given input pattern, where the number of active branches is determined by *n*^Ca^.

**Figure 3:**
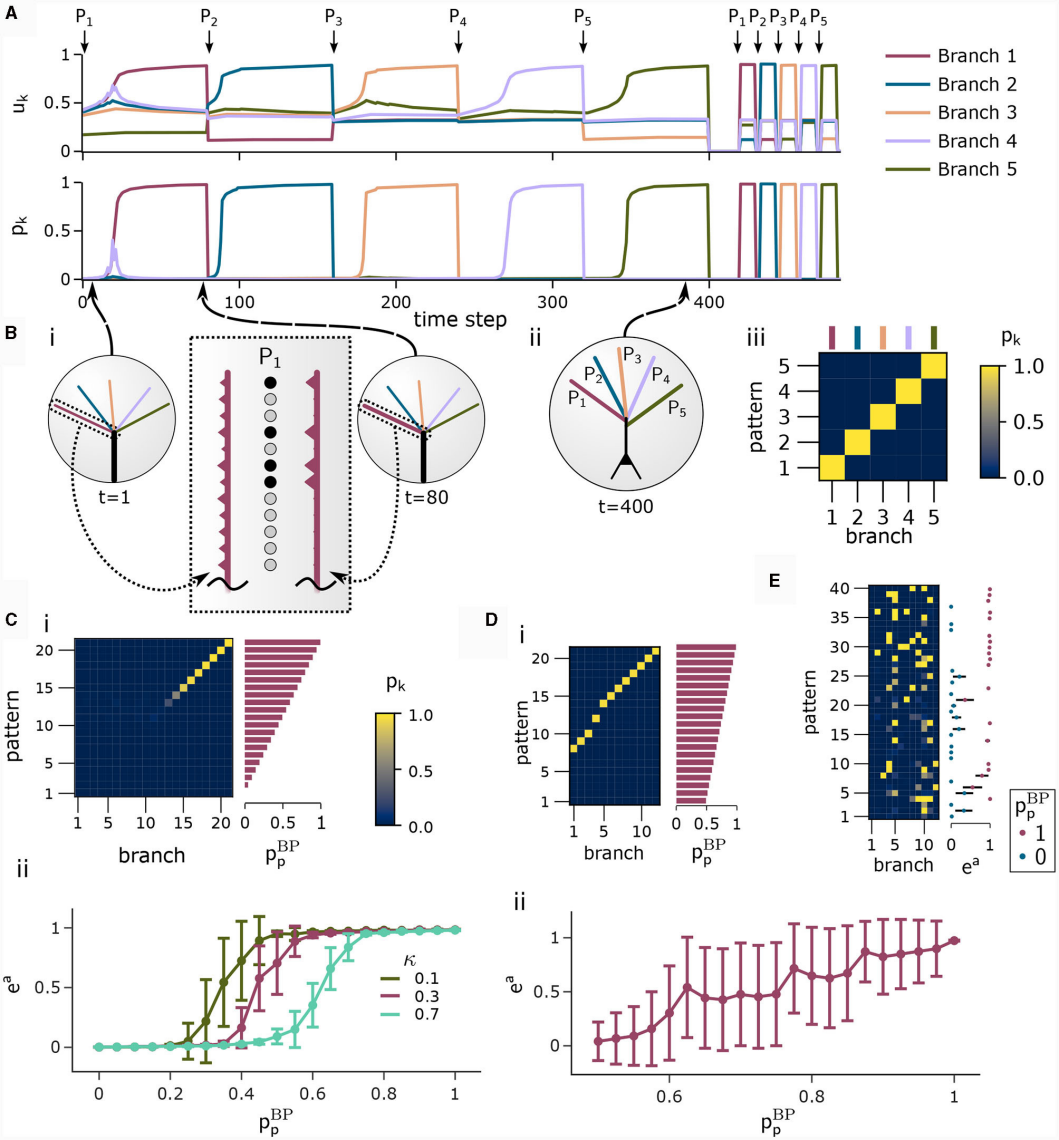
Pattern learning experiment. CAL induces synaptic cluster formation in apical dendrites. **(A)** A neuron with 5 dendritic branches sequentially associates 5 different patterns *P*_1_ to *P*_5_. (Top) The branch potential *u*_*k*_ (top) and NMDA spike probability *p*_*k*_ = σ_d_(*u*_*k*_) (bottom) for each branch *k* = 1, …, 5 are shown. Arrows indicate onset of pattern presentations. Each pattern is presented 80 times. At the end of the trial, each pattern is presented again 10 times to confirm that the functional synapse cluster still persists. **(B)** (i) Example tuning of a branch. The rectangular dashed inset shows the synaptic weights of an apical branch at the first (left) and the last (right) presentation of pattern *P*_1_. Synapse size is proportional to efficacy. (ii) After all 400 pattern presentations, every branch tuned to a different pattern. (iii) NMDA spike probability of branches in response to patterns (tuning matrix). The labeling of branches was done *post-hoc* according to the order of cluster acquisition to improve the visibility of the tuning matrix. **(C)** Pattern association depends on probability of backpropagating activtiy $p_{p}^{\text{BP}}$. (i) Tuning matrix of a single trial with κ = 0.3. Each pattern *P*_*p*_ was presented alongside samples ûBP from a fixed pre-assigned probability distribution $ℬ(p_{p}^{\text{BP}})$ with $p_{p}^{\text{BP}}$ in the interval [0, 1]. (ii) Mean (filled circle) and variance (whiskers) of apical excitation *e*^a^ for different values of κ ∈ {0.1, 0.3, 0.7} over 20 independent runs of the experiment shown in **(C)**i. **(D)** Pattern association with more patterns than branches. (i) Tuning matrix of branches for a single trial. $p_{p}^{\text{BP}}$ for each pattern *P*_*p*_ was uniformly distributed over the interval [0.5, 1]. (ii) Mean apical excitation *e*^a^ for different levels of $p_{p}^{\text{BP}}$ over 40 independent runs of the experiment shown in panel Di with κ = 0.3. **(E)** Tuning matrix in a setup with sequential pattern presentation, sparse connections, Ca^2+^ spike threshold *n*^Ca^ = 2, and more patterns than branches. The scatter plot on the right shows the apical excitation *e*^a^ caused by the presentation of the patterns after training. See main text for details.

We investigated the behavior of the rule in four scenarios. The neuron and input parameters for these experiments are summarized in Table 1. We first considered an apical arborization with 5 branches. Synaptic efficacies were initialized randomly from a Gaussian distribution with mean 0.4*w*_max_ and standard deviation 0.1*w*_max_ for *w*_max_ = 0.25. We defined five apical input patterns *P*_1_, …, *P*_5_ where each pattern represented the firing activity of an assembly of 12 afferent neurons, with 4 randomly chosen neurons being active ($x_{i}^{\text{apical}}=1$) while the remaining neurons *j* remained silent ($x_{j}^{\text{apical}}=0$). To avoid highly similar input patterns, we enforced that not more than one of the active neurons in one pattern were active in another pattern. Further details on the generation of patterns can be found in Section 4.7. The input patterns were sequentially presented as apical input *x*^apical^ (Figure 3A), i.e., pattern *P*_1_ was first presented 80 times, followed by 80 presentations of pattern *P*_2_, and so on. To analyze the association behavior of the CAL rule, each presentation was paired with a constant backpropagating activity *u*^BP^ = 1, and the CAL rule (Equation 3) was applied. Figure 3A shows the evolution of the branch potentials *u_k_* and the NMDA spike probabilities *p*_*k*_ of all branches during these presentations. One can observe that at initial pattern presentations, multiple branches increased their branch potential. After few presentations however, only one branch emerged that was strongly activated by the pattern while the other branches reduced their response. Since we defined *n*^Ca^ = 1 in this experiment, a single NMDA spike sufficed to elicit a Ca^2+^ spike. In the following, we will say that the branch is "tuned" to the pattern. Note, that due to overlap between patterns, branches depolarized in response to other patterns than the one they were tuned to, but the non-linear transfer function σ_d_ still ensures low NMDA spike probability in these cases. One example of a synapse cluster formation is shown in Figure 3Bi. In the rectangular inset, synaptic efficacies of branch 1 are compared before (left) and after (right) tuning to pattern *P*_1_ (middle). The synapses which received high input through *P*_1_ underwent LTP, the inactive synapses LTD. After all 400 pattern presentations (Figure 3Bii), each of the 5 branches tuned to one of the population patterns. This segregation of patterns can also be observed in the tuning matrix in Figure 3Biii, where we show the NMDA spike probability for each branch and pattern at the end of the experiment. Note that the activation of apical branches by input patterns is preserved after the presentation of all patterns even for patterns that have been presented at the beginning of the learning trial, see also Figure 3A (right). This continual learning capability was not explicitly defined in our objectives, but is a side-effect of the clustering of patterns onto distinct branches. We further discuss this property of the CAL rule in Section 2.6.

**Table 1:** Network and input setup for the pattern learning scenarios from different panels of Figure 3.

**Parameter**	**Experiment (panel)**			
	**1 (A)**	**2 (C)**	**3 (D)**	**4 (E)**
Apical input pattern size	12	400	400	600
Active components in apical input pattern	4	40	40	90
Number of apical input patterns	5	21	21	40
Number of apical dendritic branches	5	21	12	12
Ca^2+^spike threshold *n*^Ca^	1	1	1	2
sparse apical connections	No	No	No	Yes

We next asked how the likelihood of backpropagating activity $u_{p}^{\text{BP}}$ affects the tuning of branches. To that end, we set up a scenario in which a certain probability $p_{p}^{\text{BP}}$ was a-priori assigned to each pattern *P*_*p*_. Each time a pattern *P*_*p*_ was presented as apical input *x*^apical^, it was paired with backpropagating activity *u*^BP^ = 1 with probability $p_{p}^{\text{BP}}$ (otherwise *u*^BP^ = 0). We increased the number of patterns and branches for this experiment to 21 to obtain fine-grained results for multiple levels of *u*^BP^. We also presented the patterns in a random order, so that at each presentation, one of the 21 patterns was randomly chosen from a uniform distribution. This protocol eliminates bias introduced by the ordering of patterns. To demonstrate scaling of the update rule to larger input dimensions, we increased the pattern size to 400 input neurons, of which 40 were active in each pattern. We further increased the maximum pairwise overlap between patterns such that up to 40% of active neurons in a pattern were also active in another pattern, increasing the difficulty of discriminating patterns. Figure 3Ci shows the tuning matrix of a single run alongside the assigned probability $p_{p}^{\text{BP}}$ to each pattern *P*_*p*_. Again, it can be observed that at most one branch tuned to a pattern and that each branch tuned to at most one pattern. We observe that a certain level of backpropagating activity is required to coincide with the presentation of a pattern to allow any branch to tune to it. This is in alignment with our hypotheses from Section 2, where we emphasized the coincidence-dependence of the CAL rule. Only if apical input coincides with elevated somatic neuron activity, the formation of synapse clusters is initiated. In Figure 3Cii we show that this effect is consistent across multiple runs by plotting the average apical excitation *e*^a^ for various levels of $p_{p}^{\text{BP}}$ over 20 runs. We can see that the CAL rule reliably associates apical input patterns for high $p_{p}^{\text{BP}}$ while no association happens for low levels of $p_{p}^{\text{BP}}$. Remarkably, the probabililty $p_{p}^{\text{BP}}$ not only determines which clusters are formed, but also the magnitude of response following re-activation of the branch, which is indicated by the monotonous increase of the graph. For higher values of the dissociation coefficient κ, higher probabilities of back-propagating activity are required in order to establish a synaptic cluster. This result is coherent with the previously discussed role of parameter κ as trading off the two objectives of association and dissociation (see Section 2.2).

In the above simulations, the number of branches matched the number of different input patterns. In a third experiment, we aimed to investigate the properties of the learning rule in the case where more patterns than branches occurred, hence not all input patterns could be represented by synapse clusters on different branches. The pattern configuration was the same as in the previous experiment, with 400-dimensional input and 40 active neurons. In contrast to above experiments, we chose $p_{p}^{\text{BP}}$ in the interval of [0.5, 1] to ensure that all patterns are accompanied by a $p_{p}^{\text{BP}}$-level sufficiently high to induce tuning (as observed in Figure 3Cii). We show the result of a single trial in Figure 3Di, where the 8 patterns supported by the highest $p_{p}^{\text{BP}}$ levels induced synaptic clusters on dendritic branches, whereas some patterns with lower $p_{p}^{\text{BP}}$ did not. Again, the average apical excitation tends to increase with the backpropagating activity of the pattern, see Figure 3Dii.

We next asked whether these results also hold for a larger Ca^2+^ spike initiation threshold *n*^Ca^ and sparse connectivity to apical branches. In a fourth experiment, we used *n*^Ca^ = 2 (see Supplementary material Section S3 for a more exhaustive figure and for an experiment with *n*^Ca^ = 3), and each of the 12 branches received synapses from a sub-set of 10% of the 600 apical input neurons. We sequentially presented 40 patterns, of which we randomly chose 20 to be accompanied with *u*^BP^ = 1, the other 20 with *u*^BP^ = 0. The tuning of branches to patterns after training is shown in Figure 3E. Patterns that were presented together with high *u*^BP^ = 1 elicited significantly higher mean apical excitation (mean *e*^a^ = 0.92) upon presentation after training, compared to patterns paired with *u*^BP^ = 0 (mean *e*^a^ = 0.07). Due to the higher Ca^2+^threshold *n*^Ca^ = 2, typically two branches were tuned to a single pattern (Figure 3E). We also investigated sparse connectivity in the case of equal numbers of branches and patterns with *n*^Ca^ = 1 in Supplementary material Section S2.

### 2.4 Population-level coincidence detection via CAL

In the above experiments, we investigated the single-neuron learning properties of CAL in detail. We showed how the formation of synaptic clusters on apical dendrites depends on the coincidence of basal and apical activity. On a population level, this association principle allows a pool of neurons to detect whether the distribution of basal activity across neurons matches the expectation given a certain context indicated by apical input. Each pyramidal neuron, after coincidence-based training using CAL, can thereby act as a coincidence detector by elevating its response if apical and basal activation coincide. Such coincidence-triggered association of high-level context with lower-level stimuli was proposed as an essential principle of cortical organization (Larkum, 2013). These learned associations can cause elevated neural population response if context and low-level perceptual stimulus cohere.

**Figure 4:**
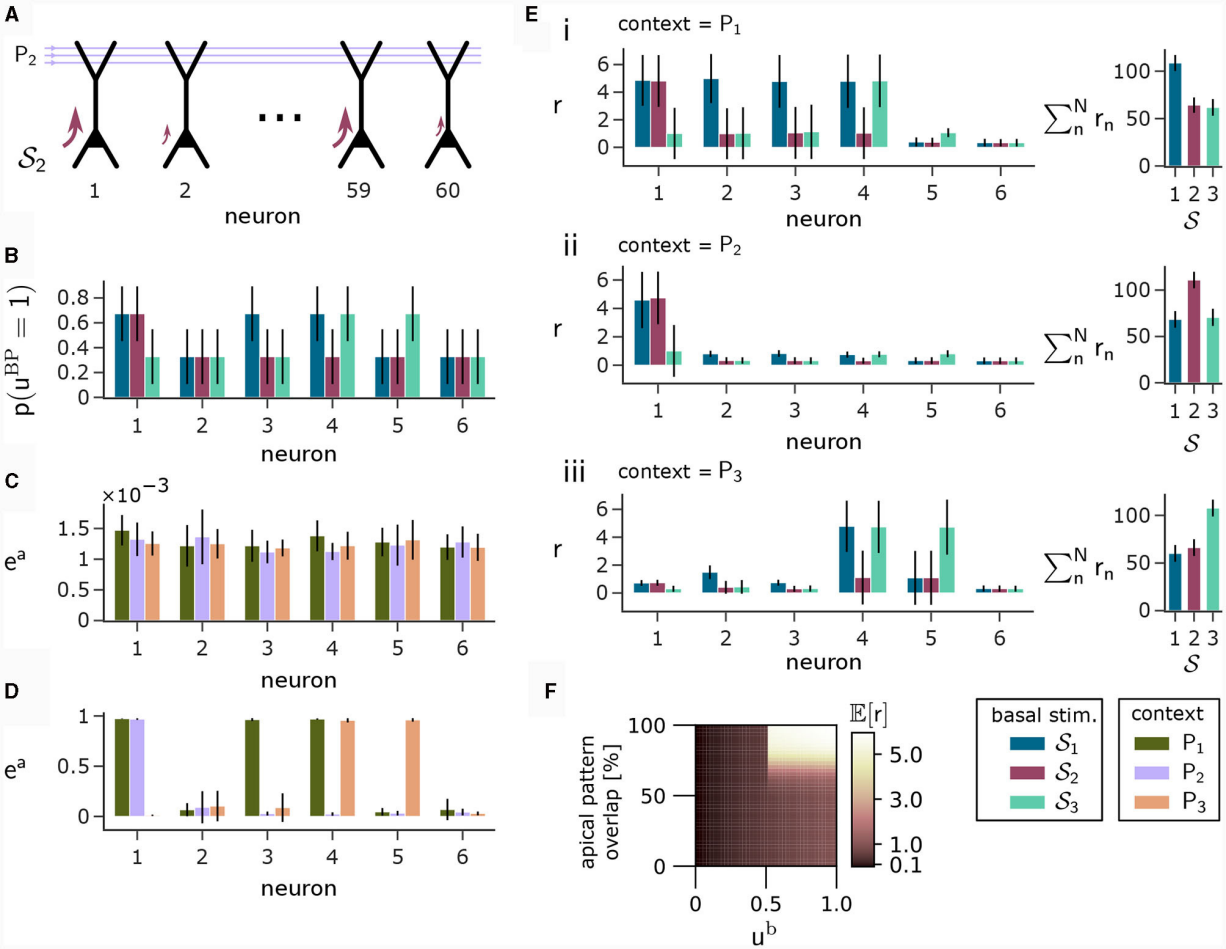
Coincidence detection in populations of pyramidal neurons. **(A)** For each context *P*_*p*_ with *p* = 1, 2, 3 we selected a sub-set of neurons receiving high basal stimulation (large arrows), whereas the other neurons received low basal stimulation. **(B)** Distribution of back-propagating activity *u*^BP^ resulting from basal stimulation distributions. Bars show the mean, whishkers show the variance. **(C)** Apical excitation *e*^a^ per neuron for each context before training. **(D)** Same as **(C)**, but after training with CAL. **(E)** Output firing rate *r* during presentation of contexts together with some basal stimulation distributions after training. (i) left: Neuron output rate *r* for cued context *P*_1_ together with basal stimulation distributions S_1_, S_2_ and S_3_. right: summation of output rates over the neuron population. If the basal stimulation distribution (S_1_) matches the cued context (*P*_1_), the population response is highest. (ii, iii) Same as (i), but for contexts *P*_2_ and *P*_3_ respectively. **(F)** Coincidence detection mechanism of a single neuron with a single branch in detail. The heatmap shows the expected output rate *r* (see Equation 6) for different levels of overlap between a presented context pattern with the associated context pattern of the branch on the y-axis and different levels of *u*^b^ on the x-axis. Only if high activity levels in both apical sites coincide, the neuron responds with a high output rate. The step-like increase in the expected output rate 𝔼[*r*] along the x-axis appears since a Ca^2+^ spike can be triggered only when the basal potential *u*^b^ exceeds the threshold *θ*^b^ = 0.5 (see also Equation 5).

To illustrate this principle, we conducted an experiment in which we simulated a pool of 60 model pyramidal neurons, subject to plasticity according to our CAL rule (neuron and input parameters are summarized in Table 2). Each neuron should thereby learn under which context elevated basal activity was expected. The experimental procedure is illustrated in Figure 4A: Upon presentation of a contextual pattern *P*_*p*_ to the apical dendrites, basal activations *u*^b^ were sampled from a specific stimulation distribution S_*p*_. For each S_*p*_, we randomly selected a sub-population of 20 neurons (in the example from Figure 4A, among others, neurons 1 and 59 for context 2) receiving high basal stimulation, sampled from a Gaussian probability distribution with high mean 0.7. All other neurons received low basal stimulation during this context, sampled as well from a Gaussian distribution but with low mean 0.3. For each different contexts *P*_1_, *P*_2_, *P*_3_, a different sub-set of neurons was selected as strongly stimulated. The back-propagating activity *u*^BP^ resulted then from thresholding *u*^b^, via *u*^BP^ = Θ(*u*^b^−θ^b^), as described in Section 2.2, with a threshold θ^b^ = 0.5. We plot the resulting probability *p*(*u*^BP^ = 1) for the first 6 neurons in Figure 4B. Before the synapses on apical branches were trained, the apical excitation *e*^a^ for each neuron and context were almost uniform, see Figure 4C. After training, synapse clusters to contexts were formed on apical compartments of neurons, in which high basal activity was expected according to stimulation distribution S_*p*_ (Figure 4D, compare with Figure 4B) for context *p*.

**Table 2:** Network and input setup for the population coincidence detection experiment from Figure 4.

**Parameter**	**Value**
Apical input pattern size	400
Active components in apical input pattern	40
Apical dendritic branches per neuron	10
Number of different contexts	10
Neuron population size	60
Strongly stimulated neurons per context	20

After learning, we tested the coincidence detection capabilities of the neurons. To that end, we held the context pattern fixed whilst presenting basal activation levels according to the distributions from all contexts, see Figure 4E, subpanels i to iii, compare with Figures 4B, D. Firstly, we can observe that the simultaneous presentation of context *P*_1_ (see Figure 4Ei) at the apical site and stimulation distribution S_1_ at the basal site results in high output rate *r* for neurons 1, 2, 3 and 4, due to the matching of apical and basal activity. Secondly, by presenting context *P*_1_ together with a mis-matching basal stimulation distribution S_2_ or S_3_, we can only see high neuron output *r* in the context-overlapping neurons. For example, neuron 1 was strongly stimulated in distributions S_1_ and S_2_, hence it elicits high firing rate upon stimulation according to both of these distributions if context *P*_1_ is cued. In contrast, neuron 4 underwent only low stimulation via S_2_, resulting in low firing rate if *P*_1_ is simultaneously presented with samples from S_2_, indicating a mismatch between expected and real basal stimulation. On a population level, this coincidence detection is visible on the right hand side of (i–iii) in Figure 4E, where the total population activity is highest if the basal stimulation during presentation of context pattern *P*_*p*_ matches the basal stimulation distribution S_*p*_ applied during training. This way, the neurons can learn to perform coincidence-detection through the coincidence-based nature of the CAL learning rule and the coincidence-triggered amplification from Ca^2+^ spikes, see Figure 4F.

### 2.5 The CAL rule enables context-feature association

We have shown in the previous section that the CAL rule is well suited to align two separate streams of information: internal contextual information arriving at the apical and stimulus-driven information arriving at the basal compartment. We wondered whether the model can utilize such context-association to solve multi-task classification problems.

**Figure 5:**
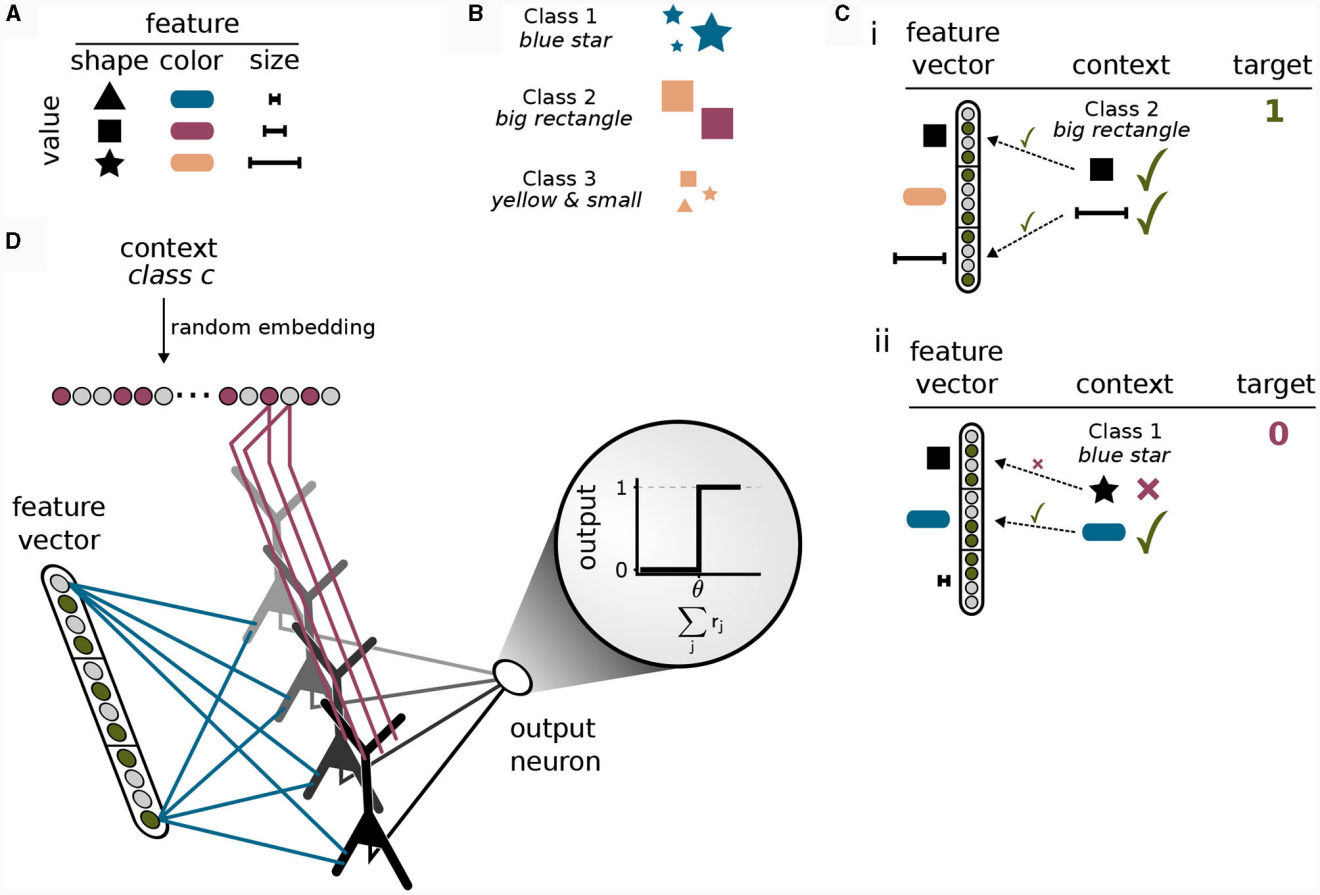
Solving a context-dependent classification task by binding contexts to features. **(A)** Features can be interpreted as different properties of an object, where each feature is instantiated in one of its values. **(B)** Classes are defined a priori by specific combinations of features. Features not included in the class definition (for example “size” for class 1) should be ignored. **(C)** Positive and negative samples. (i) In a positive sample, all feature values required for the class given as context are contained in the feature vector. (ii) If one or more feature values in the feature vector deviate from the required values given by the class in the context, the sample is negative and the target output is 0. **(D)** Illustration of the network model for the context-dependent feature association task. The model consists of an input layer that is fully connected to the basal dendrites of a single hidden layer of pyramidal neurons followed by a fully-connected output layer consisting of a single threshold neuron. The input to the apical dendrites is the context vector, encoding a class identifier via a random embedding. The task of the network is to detect whether the class represented by the context is present in the feature vector or not. The network can only solve the task by correctly integrating bottom-up and context input since both are required to provide a correct answer. Synaptic efficacies between red axons and apical dendrites of pyramidal neurons are trained by CAL, whereas synaptic efficacies between blue axons and basal dendritic site are trained using the Krotov-rule (see main text).

To this end, we considered a simple multi-task classification problem which we termed the context-dependent feature association (CDFA) task set. In the CDFA task set, the input arriving at the basal dendrites represents some encoded perceptual low-level stimulus, whereas the context holds information about an attended object class (high-level context). The goal of this task set is to successfully determine the presence or absence of the attended object class within the perceptual stimulus. As a guiding example, we assume that the sensory stimuli encode the values of visual features like shape or color (Larkum, 2013). Each feature can take on one of a number of discrete values. For example, the shape can be a triangle, a square, or a star, the color can be blue, red, yellow, etc., see Figure 5A. We define an object class as a certain combination of feature values, where some features are irrelevant. For example, the object class “blue star” is defined as inputs where the feature “color” is “blue” and the feature “shape” is “star”, see Figure 5B. In the CDFA task set, the input to a network is given by a feature vector as the bottom-up input together with context information about an object class and the task is to output 1 if the feature values contained in the input match those of the indicated object class (where don't care features of the object class should be ignored) and 0 otherwise, see Figure 5C. We refer to samples where the target is 1 as 'positive', and to samples where the target is 0 as “negative”. This setup defines a multi-task classification problem, where each task is defined by one object class (the context). In each individual task, one thus has to determine whether a specific set of feature values is present in the feature vector (while ignoring some of the features).

We considered a network consisting of one layer of 60 model pyramidal cells, each with 10 dendritic branches (see Figure 5D and Table 3). The pyramidal cell layer projected to a single readout neuron which should produce the correct output according to the CDFA task. The output *y* of the readout neuron was given by


(14) $$\begin{array}{l} y=\Theta \left(\underset{j}{\sum }r_{j}-\theta \right), \end{array}$$

where θ represents a learned threshold, *r*_*j*_ denotes the output of pyramidal neuron *j* in the pyramidal layer, and Θ denotes the Heaviside step function. Similar to the population coincidence detection experiment from the previous section, this threshold should allow the network to distinguish between a match or a mis-match of context and sensory stimulus. In order to facilitate unsupervised learning at basal compartments (see below), the neurons in the pyramidal layer implemented a K-winner-take-all (k-WTA) structure: The basal membrane potential $u_{j}^{\text{b}}$ of a neuron *j* was set to one if it was among the *K* largest potentials in the layer, otherwise 0 (see *Methods*; we used *K* = 6 in our simulations).

**Table 3:** Network and input setup for the context-dependent feature association experiment.

**Parameter**	**Value**
Apical input pattern size	60
Active components in apical input pattern	9
Apical dendritic branches per neuron	10
Neuron population size	60

The pyramidal cells received as basal input the visual features encoded in the activity of 600 bottom-up input neurons, see *Methods* Section 2.5. The apical input to the neurons consisted of the context object class, each encoded as a 60-dimensional random sparse class vector. Note that the object class encoding does not entail any direct information on relevant features. One can consider it as a name, such as "Tiger." This name does not give us a-priori information about the relevant features which could be “texture=stripes”, “color=orange”, “shape=cat-like” (Larkum, 2013).

The CAL rule only defines weight updates Δ*w*_*lj*_ (see Equation 12) for apical synapses, not for the synaptic weights **v** in the basal compartment. To train networks of such neurons, a synaptic plasticity rule for the basal weights **v** is required as well. We wondered whether local unsupervised learning of basal weights together with the CAL rule for apical weights and simple local supervised adaptation of the readout neuron would suffice to learn the CDFA task. To that end, we trained the basal weights **v** with the biologically plausible local Hebbian learning rule from Krotov and Hopfield (2019) (the “Krotov-rule”, see *Methods*). This unsupervised Hebbian learning was performed in a first step to establish a suitable feature representation in the pyramidal layer. Afterwards, associative adaptation of apical weights through the CAL rule and local supervised learning of the readout was performed (see *Methods*). Training of the readout neuron was performed through gradient descent, where only the threshold θ was adapted. Note that only the readout threshold θ was trained in a supervised manner. Hence, supervised learning was local and restricted to the single readout neuron.

**Figure 6:**
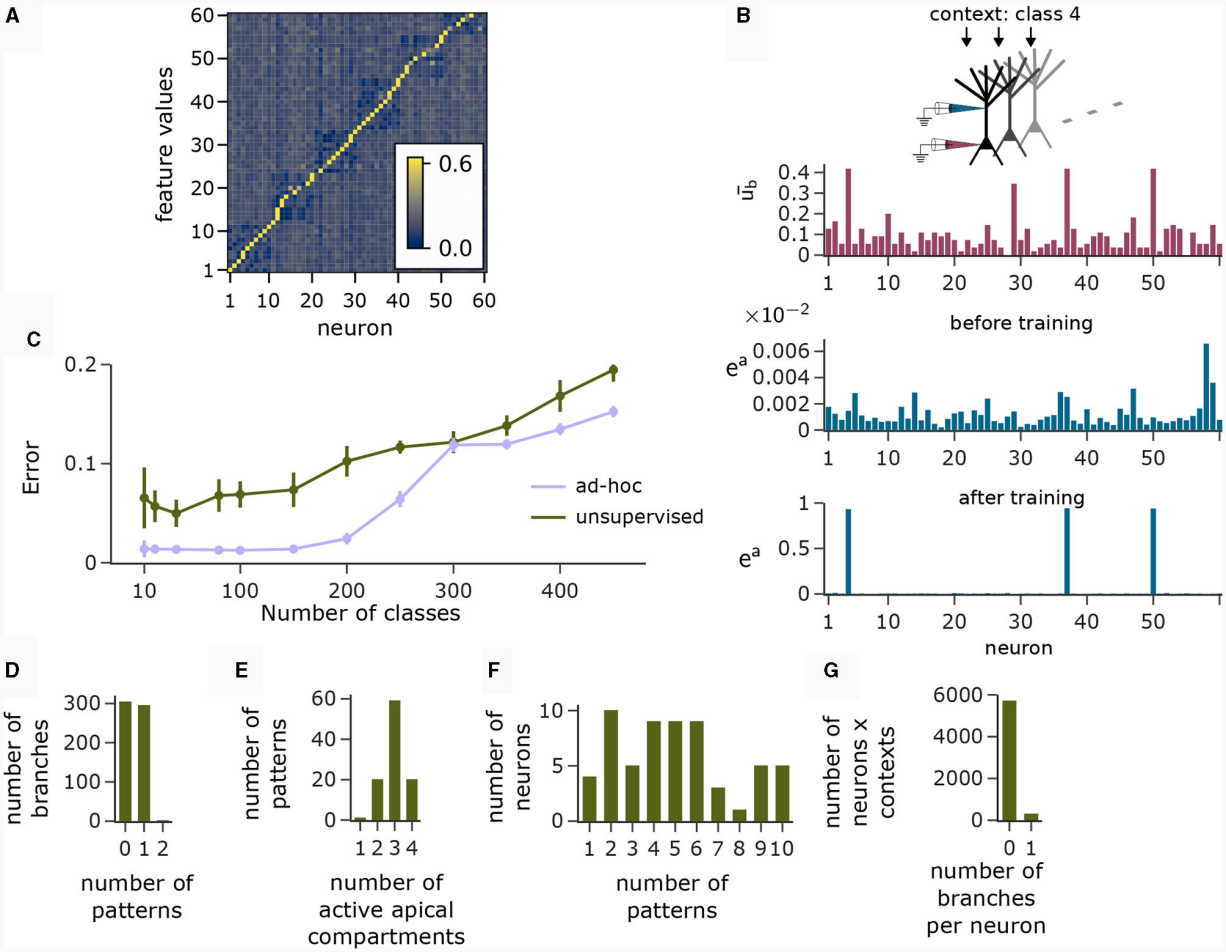
Analysis of network activity in the CDFA task. **(A)** Pairwise cosine similarity between basal weights and feature value encodings of the CDFA task after unsupervised learning with the Krotov rule. Neurons are sorted by the feature index at which their basal weights show maximum similarity. **(B)** CAL exploits statistical correlations between context inputs and basal activity. Red bars: average basal output $\bar{u^{\text{b}}}$ (induced by bottom-up feature vectors, after k-WTA was applied) across all pyramidal neurons in one specific object context. Blue bars: apical excitation of the same neurons in the same context before (top) and after (bottom) training. **(C)** Classification error as a function of the number of object classes for unsupervised training (green) and an *ad-hoc* setting (violet) of basal weights. **(D)** Histogram showing the number of branches which are tuned to 0, 1 or 2 patterns. Nearly every branch tuned to either one or no context pattern. **(E)** Histogram showing the number of patterns that activate a certain number of neurons (i.e., neurons with *e*^a^>0.5). Most contexts activate 3 neurons, which is expected given the fact that each class is uniquely identified by 3 distinct feature values. **(F)** Histogram showing the count of neurons that are activated by a specific number of contexts. **(G)** Histogram showing how many branches per neuron are activated over all contexts. No context activates more than 1 branch on any neuron and the activation of neurons is generally very sparse. Panels B and D-G show statistics for the experiment with 100 object classes.

The motivation behind k-WTA-like Hebbian learning of basal synapses was that this learning paradigm might adapt basal compartments to become detectors for specific feature values. Figure 6A (right), shows the cosine similarity between the learned basal weight vectors and all 60 feature value encodings (six features with 10 values each) after applying the Krotov rule to the basal synapses in the pyramidal layer during the presentation of 1000 randomly chosen input feature vectors. The network learned an approximately orthogonal representation of independent features in its basal weights. Hence, each neuron functioned as a detector for a specific feature.

Using such representations, we asked whether the CAL rule was able to exploit statistical correlations between top-down object class representations and basal activity to associate input features with object classes and whether simple training of the readout could solve the CDFA task. To this end, we presented 250 training samples per class to the network, each consisting of a feature vector **f** and one object class context *c* for 100 different object classes. During this presentation, the object class identifier provided as context matched to the feature vector (target ŷ = 1) in half the cases, and they did not match in the other half (ŷ = 0).

The red bars in Figure 6B show the average basal output *u*^b^ across all pyramidal neurons during the occurrence of a specific context cue after unsupervised training of basal weights. One can observe that neurons 4, 37, and 50 were particularly strongly activated in that context, indicating object class-relevant features. The upper and lower blue bar charts show the apical excitation of the same neurons for that context before and after training respectively. The CAL rule has boosted apical activation of those neurons with strong basal activity. If a feature coincided with a context to which it had previously been associated, the corresponding neuron exhibited increased activity, thus indicating a match between the context and the bottom-up input. Thus, the CAL rule has turned neurons from feature detectors into coincidence detectors, allowing the network to detect whether the class-relevant features are present in the basal input or not. This association through the CAL rule was due to the correlation of the object context with the particular feature it detected in the basal input. This mechanism worked despite the presence of “false” correlations in the data due to negative examples where the context object class did not match the input feature vector (compare with Figure 5Cii), although the CAL rule had no information on whether the current data sample is positive.

We next analyzed the representation of context patterns on dendritic branches of modeled pyramidal neurons. As in the pattern association experiment above, each branch tuned to either one or no context pattern (Figure 6D). Hence, context patterns were clustered on dendritic branches. Further, we found that the majority of patterns activated 3 apical compartments within the whole pyramidal layer (Figure 6E). Since each object class was determined by 3 feature values in our task setup, this shows that the CAL rule established an efficient association between object classes and features. As feature values can appear in different object classes, neurons were in general activated in varying contexts, see Figure 6F. Overall, the activation of branches in response to contexts was very sparse. In Figure 6G, we show a histogram over the number of active branches in a given neuron for a given context (histogram taken over all neurons and contexts). We observe that no context activated more than a single branch on each neuron, which is a natural consequence of setting the Ca^2+^ spike threshold *n*^Ca^ to 1.

Using the population coincidence mechanism installed by the CAL rule (also demonstrated in Section 2.4), the readout neuron was able to solve the CDFA task simply by locally adjusting its threshold. This was possible since elevated activity in the pyramidal layer indicated a match between the contextual object class and the feed-forward features. With 100 object classes, the classification error of the network was 6.89 ± 1.32%. We report the classification error of the model for various numbers of classes in Figure 6C. The more classes, the more dendritic resources are required to establish context-feature associations, resulting in exhaustion of capacity for higher numbers of classes and a consequential increase in mis-classifications. A simple calculation (see Supplementary material Section S4) shows that our network setup allows for the storage of 200 patterns in expectation. Nevertheless, Figure 6C shows that performance degrades gracefully for up to 400 patterns. As a baseline, we also considered the behavior of a network with an “optimal” *ad-hoc* defined set of basal weights, in which the basal weight vector of each neuron was manually set to one of the feature values. This induced a perfectly orthogonal representation of features in the basal activity of neurons. We can see, that the fuzzy feature representation from the unsupervised learning algorithm for basal weights adds a layer of difficulty compared to the *ad-hoc* setting of input weights that perfectly decodes the feature values. This is due to the lack of representation of certain feature values in the pyramidal layer in the fuzzy representation, resulting in reduced separability of the classes. We also tested a modified version of the Krotov rule (termed “Krotov+” in the following), which is still fully unsupervised but less biologically plausible (see *Methods*). This rule achieved a nearly perfect orthogonal representation of input feature values. With this more powerful unsupervised learning, the classification error was 1.44 ± 0.3%.

### 2.6 The CAL rule enables continual learning

Training artificial neural networks on multiple tasks sequentially with backpropagation can result in “catastrophic forgetting” (McCloskey and Cohen, 1989). It has been suggested (Cichon and Gan, 2015; Limbacher and Legenstein, 2020; Sezener et al., 2021; Acharya et al., 2022) that biological neurons exploit active dendritic properties to avoid over-writing of previously learnt information. As we showed already in the pattern association experiment in Section 2.3, CAL can effectively distribute newly learnt associations to untuned branches, protecting branches previously engaged in forming synaptic clusters from re-tuning. We tested whether this property can be exploited in an incremental learning scenario of the CDFA task, where new tasks are learned on top of previously learned ones in a continual fashion. As before, a task consisted of one object class representation which was provided as context to the apical branches and the goal was to classify whether the current input feature vector was consistent with this object class.

We trained the same network as in Section 2.5 in a three-stage process: In the first stage, 1, 000 random feature vectors were generated to train the basal weights in an unsupervised manner with the Krotov rule as before. This established a stable feature representation in the basal weights. In the second stage (“pre-training”), the basal weights were frozen and we trained the network on 40 different tasks while the CAL rule as well as training of the readout threshold was enabled. During this stage, the network could learn to distinguish between positive examples and negative examples by thresholding the total summed activity in the pyramidal neuron layer. In the third stage, this threshold θ was frozen and the network (with CAL still enabled) was trained on 8 new tasks sequentially. Each of these tasks contained an equal number of positive and negative examples for a certain new object class that has not been shown before.

**Figure 7:**
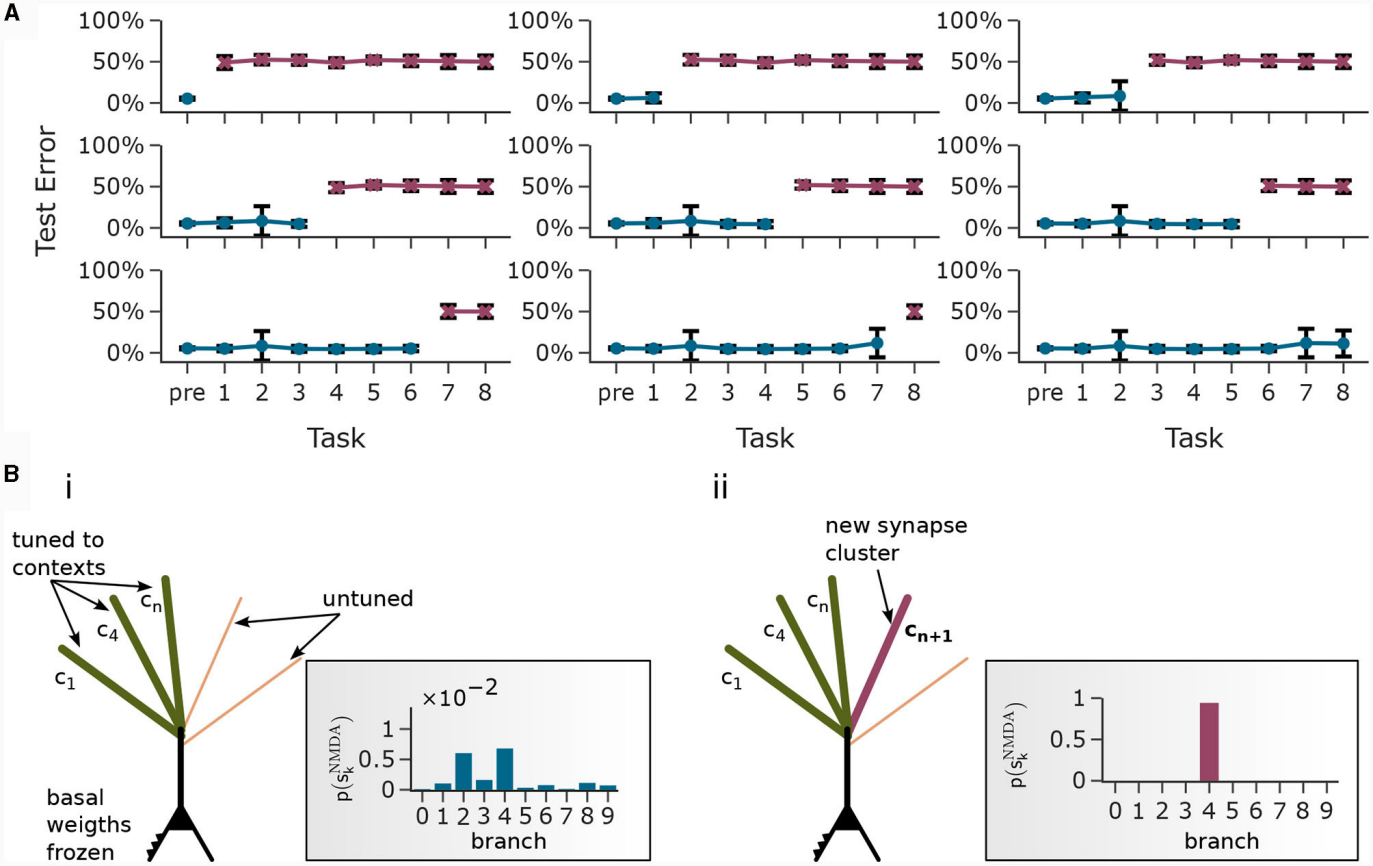
Continual learning through context-association learning. **(A)** Results on CDFA in a continual learning setup averaged over 20 runs. We pre-trained the readout threshold θ together with the first 40 tasks, then froze θ and trained continually on 8 more tasks. Each subplot shows test accuracy on all tasks after pre-training followed by continual learning on a certain number of tasks. Bars show standard deviation. **(B)** Acquisition of a new branch by an example pyramidal neuron. (i) Some apical dendritic branches already formed synaptic clusters to contexts of previous tasks, the rest were untuned (schematic presentation). Rectangular inset: Upon presentation of the new context, all branches exhibit low NMDA spike probability. (ii) After training on the new task, one particular branch formed a synaptic cluster responding to this new context.

In Figure 7A, each plot was generated after training the network on a new task. For example, the first plot (top left) shows the test error on all tasks directly after pre-training. Naturally, the network was unable to perform well on the upcoming tasks which it has not seen before. The second plot shows the test error on all tasks after pre-training followed by training on task 1. The other plots follow the same scheme. We observe that the model has learned new classes incrementally and at the same time maintained low test error on previously trained classes. Note that the readout was no longer trained in this phase. To do so, the network exploited statistical properties of the data. Only the neurons with strong basal activation during presentation of a new task tuned to the new context. CAL implements this incremental association by forming synaptic clusters on previously unused branches, see Figure 7B. Input feature vectors that are consistent with the object class lead to enhanced activity in the pyramidal layer due to the modulation of basal activity by the apical dendrites. This enhanced layer activity is readily detected by the readout with the previously learned threshold. After training on all tasks, we obtained a test error on all samples from all test sets of 6.81%±10.54% if basal weights were trained with the Krotov rule and 2.11%±6.55% if basal weigths were trained with Krotov+ (see Supplementary material Section S5 for a detailed figure). We explain the performance gap by the cleaner separation of independent feature values by the Krotov+ learning rule. Note, that the basal weights are trained before the first task and held fixed over the course of incremental task learning, which forces the network to use only the initially learned internal feature representation.

## 3 Discussion

In this article, we investigated what types of synaptic plasticity rules at apical dendrites could give rise to a computational function that was proposed to be central to the function of layer 5 pyramidal cells (Larkum, 2013): The association of top-down contextual activity patterns with bottom-up sensory features. Methodologically, we applied a top-down approach by formulating the learning objectives as a loss function and deriving the corresponding plasticity rule from them. The resulting CAL rule thus performs gradient descent on the proposed loss.

Several studies have shown that the computational capabilities of neurons and neural networks can be significantly extended when moving from the traditional point neuron model to models that incorporate the nonlinear dynamics of spatially extended neurons. This even holds for highly reduced models with only two (Ferrand et al., 2023) or three (Quaresima et al., 2023) distinct compartments. Our model is slightly more fine-grained as it features multiple apical dendritic branches. The computational role of these branches in our model is to assure reliable storage of all context patterns while avoiding spurious activations of the apical region through random context patterns, see also (Legenstein and Maass, 2011; Limbacher and Legenstein, 2020). Consider the case that a single branch would be used in experiment 1 (Figure 3A). As the apical region should be activated by all presented patterns *P*_1_ to *P*_5_ after learning (as illustrated on the right of Figure 3A), a large number of synapses from each of these patterns would have to be potentiated. In that case, most random context patterns would activate the apical region. This is true both for a continual learning setup as in Figure 3A where patterns are presented sequentially, and in a setup where patterns are presented in an intermixed manner (Figures 3C, D). In contrast, the branch nonlinearity assures that partial synaptic activation of one or several dendritic branches leads to weak apical activation, see Figures 1B, 4F.

When a single NMDA spike can elicit a Ca^2+^ spike (*n*^Ca^ = 1), we found that each branch typically stores one context patterns. Hence, the number of patterns that can be reliably stored is given by the number of branches. One can observe this well in Figure 6C, where the error increases strongly for more than 200 object classes in the *ad-hoc* “optimal” basal weight setting. At this number, it is expected that all branches are used to store context patterns in our network. Our simulations for *n*^Ca^>1 (Figure 3E and Supplementary material Section S3) indicate that the capacity is larger in this case. For *n*^Ca^ = 2, the number of associated patterns (20) exceeded the number of branches (10), but the model still showed significantly enhanced activity for patterns that were previously accompanied by strong back-propagating activity, compared to patterns with low back-propagating activity. We leave the study of the model capacity in this case for future work.

From the biological perspective, the CAL rule can be interpreted as a rule that combines synapse-local information with branch-local information (branch potential and branch-wide NMDA spikes) and two types of more global signals: somatic activity and global Ca^2+^events. An essential ingredient in the CAL rule is the dependence on NMDA spikes. In particular, synapses at dendritic branches with frequent NMDA spikes tend to become potentiated, see line 2 of Equation (13), benefiting the formation of synaptic clusters. A role of NMDA spikes in LTP has also been established in experiments (Gambino et al., 2014).

The CAL rule predicts that synapses should not be further potentiated when Ca^2+^ spikes appear. This behavior avoids that too many branches tune to the same contextual patterns, which would waste dendritic resources and reduce synaptic clustering. To the best of our knowledge, such plasticity has not been reported for apical synapses yet. Hence, it can be viewed as an experimental prediction of this work. Other predictions that arise from the CAL theory are as follows: First, neurons tune apical dendritic branches to certain contexts in which they previously showed increased levels of activity. Second, the back-propagating action potential acts as a trigger for this mechanism and its absence during depolarization of the apical dendritic compartment leads to LTD. We note that a similar effect has been observed in (Sjöström and Häusser, 2006). And third, neurons recruit untuned apical dendritic branches in new contexts in order to associate a new context in a manner that does not interfere with previously learned associations. The segregated activation of dendritic branches has been observed in mouse motor cortex (Cichon and Gan, 2015). However, its sequential recruitment has not been demonstrated so far. In general, we do not expect that the exact behavior of the CAL rule can be demonstrated in experiments. Nevertheless, it will be interesting to see which aspects of its objective can be found in apical synapses.

A central objective of the CAL rule is to cluster functionally related synapses on dendritic branches. We showed that this principle enables continual learning in networks of pyramidal cells. Models for clustered synaptic plasticity have been developed by several authors (Poirazi and Mel, 2001; Wu and Mel, 2009; Legenstein and Maass, 2011; Limbacher and Legenstein, 2020; Bicknell and Häusser, 2021). These works however did not consider synaptic clustering in the context of associative learning as we did. The work by Rao et al. (2022) developed a plasticity rule termed Dendritic Logistic Regression that enables the apical compartment to predict somatic activity. This objective is somewhat related to our association objective, although we did not attempt to produce a precise probabilistic prediction as in Rao et al. (2022). The authors of this paper also did not consider a clustering objective, hence activity in their model for a given apical activation is distributed over all branches. If the rule is used to associate contextual patterns, this leads to interference of these patterns and hinders continual learning. See Supplementary material Section S6 for a comparison between Dendritic Logistic Regression and the CAL rule. Plasticity of apical dendritic synapses has also been proposed as a mechnism to implement backpropagation in pyramidal neurons (Schiess et al., 2016). This implements some form of supervised learning which is a quite different objective from the associative objective proposed in this work. A local dendritic learning rule for supervised learning was proposed in Sezener et al. (2021) in combination with dendritic gating to allow for continual learning. In contrast to the CAL rule, where the goal is to associate top-down context signals with basal activity, this model focuses on learning some target output. Phenomenological models for plasticity in pyramidal cells with dendrites have been proposed and analyzed recently in several studies (Bono and Clopath, 2017; Ebner et al., 2019; Wilmes and Clopath, 2023). It was shown in Bono and Clopath (2017) that due to different local properties and in particular different levels of attenuation of the backpropagating action potential at different dendritic sites, multiple synaptic plasticity mechanisms can coexist in single cells. Ebner et al. (2019) studied plasticity at distal apical dendrites in particular. Interestingly, their model predicts that plasticity is gated by coactive basal and apical activity.

We showed that via the CAL learning rule, apical dendritic branches can tune to context patterns, allowing the neuron to amplify its response if the same contextual pattern is presented again. This way, certain contextual patterns can activate sub-networks within a network and allow context-dependent processing of input, similar to a previously proposed algorithm for multi-task learning in artificial neural networks (Masse et al., 2018), but in an unsupervised manner.

In conclusion, we have shown that context-association learning in a simpified pyramidal cell model is possible with a synaptic plasticity rule that utilizes the main experimentally reported neuron-internal signals. We have further shown that the resulting contextual amplification of network activity implements a coincidence detection mechanism that signals whether sensory input features match the contextual top-down input. This allows networks to learn to solve multi-task problems and enables continual learning.

## 4 Methods

### 4.1 Details to neuron model

#### 4.1.1 Apical branch non-linearity

The non-linear transfer function to obtain *p*_*k*_ from *u_k_* (Equation 2) is defined by a generalized Richards function of the form:


(15) $$p(s_{k}^{\text{NMDA}}|u_{k})=\sigma_{\text{d}}(u_{k})=A+\frac{K-A}{\left(C+e^{-B(u_{k}-D)}\right)}.$$

Parameters were chosen as *D* = 0.7, *B* = 20 and *C* = 1. We define $\sigma_{\text{d}}\left(0\right)\overset{\text{def}}{=}0$ to obtain $A=\frac{K}{1-C-e^{BD}}=-8\cdot 10^{7}$. We also set $\sigma_{\text{d}}\left(1\right)\overset{\text{def}}{=}1$ to obtain *K* = 1.0025. The output of σ_d_(*u_k_*) is clipped in the interval [0, 1]. Note, that by setting parameter *D* = 0.7, we get σ_d_(0.7) = 0.5. We chose this parameter to ensure that the NMDA spike probability *p*_*k*_ < 0.5 if the active neurons in the presented *x*^apical^ have less than 70% overlap with the active neurons of the pattern branch *k* is tuned to. The derivative of this function is given by


(16) $$\begin{array}{l} {\dot{\sigma }}_{\text{d}}\left(x\right)=\frac{B\left(K-A\right)e^{-B\left(x-D\right)}}{{\left(e^{-B\left(x-D\right)}\right)}^{2}} \end{array}$$

#### 4.1.2 K-winner-take-all activation

We modeled layer-wise lateral inhibition using a simple binary K-winner-take-all (k-WTA) activation. Let H^*K*^ be the set of *K* highest neuron activations $u_{j}^{\text{b}}$ of a hidden layer with neurons *j*. The k-WTA function for neuron *j* in this layer is then defined by


(17) $$q_{j}=\{\begin{array}{ll} 1, & \text{if }u_{j}^{\text{b}}\in {ℋ}^{K}\\ 0 & \text{otherwise}. \end{array}$$

Subsequently, *q*_*j*_ was used as laterally inhibited replacement of *u*^b^ in the calculation of the apical Ca^2+^ spike *S*^Ca^and the neuron output rate *r*.

### 4.2 Details to learning rule visualizations

For the visualizations of CAL in Figure 2A, we calculated the expected value of the weight updates for a model with a single branch, where an apical input pattern of size 12 with 4 active components was shown. Parameters were set to κ ∈ {0.1, 0.3, 0.6}, λ = 0.33, λ_reg_ = 4, *w*_max_ = 1/4 and *n*^Ca^ = 1. For Figure 2B, the same parameters were used, except for the number of branches, which was 2 and κ = 0.3. The starting points for the four trajectories in panel D were $\left({\bar{w}}_{1},{\bar{w}}_{2}\right)=\left(0.1,0.08\right),\left(0.08,0.12\right),\left(0.2,0.16\right),\left(0.2,0.18\right)$. The black lines show the trajectories of the weights for 2, 000 evaluations with a dendritic learning rate of 0.08. We did not sample dendritic NMDA-spikes, but rather used the expectations of weight updates to obtain smooth trajectories. The arrow lengths *d* in the phase plane analysis are a function of the logarithm of the norm of Δ**w**, given by $d=1+\text{ln}(\text{maximum}(|\Delta w|,10^{-6}))-d_{\text{min}}$ with $d_{\text{min}}=\text{min ln}(\text{maximum}(|\Delta w|,10^{-6}))$ over all obtained |Δ**w**|. |·| thereby denotes the Euclidean norm, maximum(*x, y*) the maximum of *x* and *y*, and min the global minimum.

### 4.3 Derivation of the context-association learning (CAL) rule

Our local loss function (from Equation 8) is defined as


$${ℒ}_{\text{CAL}}=u^{\text{BP}}A+\lambda u^{\text{BP}}C+\kappa (1-u^{\text{BP}})D,$$

where κ is a balancing constant. We perform optimization on this loss function via gradient descent. In the following, we derive the gradient


(18) $$\frac{\partial {ℒ}_{\text{CAL}}}{\partial w_{lj}}=u^{\text{BP}}\frac{\partial A}{\partial w_{lj}}+\;u^{\text{BP}}\frac{\partial C}{\partial w_{lj}}+\kappa (1-\;u^{\text{BP}})\frac{\partial D}{\partial w_{lj}},$$

starting with dissociation term D:


$$\begin{array}{l} \;D=\sum_{k}\sigma_{\text{d}}(u_{k})\\ \;=\sum_{k}\sigma_{\text{d}}(\sum_{i}x_{i}w_{ki})\\ \Rightarrow \frac{\partial D}{\partial w_{lj}}={\dot{\sigma }}_{\text{d}}(\sum_{i}x_{i}w_{li})x_{j}\\ \;={\dot{\sigma }}_{\text{d}}(u_{l})x_{j}. \end{array}$$

For association loss A, we obtain


$$\begin{array}{l} \;A=\text{max}\left(n^{\text{Ca}}-\sum_{k}s_{k}^{\text{NMDA}},0\right)\\ \Rightarrow \frac{\partial A}{\partial w_{lj}}=-\frac{\partial s_{l}^{\text{NMDA}}}{\partial w_{lj}}\Theta \left(n^{\text{Ca}}-\sum_{k\in K}s_{k}^{\text{NMDA}}\right) \end{array}$$

We cannot directly compute the derivative $\frac{\partial s_{l}^{\text{NMDA}}}{\partial w_{lj}}$, therefore we approximate it by the derivative of the NMDA spike probability


(19) $$\frac{\partial p(s_{l}^{\text{NMDA}})}{\partial w_{lj}}=x_{j}{\dot{\sigma }}_{\text{d}}(u_{l}).$$

We found, that ${\dot{\sigma }}_{\text{d}}\left(u_{l}\right)$ gets very small in regions of low branch potential *u*_*l*_, resulting in very small weight updates if the branch responds with low *u*_*l*_ to a given input *x*^apical^. Hence, we add a small constant ϵ = 0.08 resulting in


(20) $$\frac{\partial A}{\partial w_{lj}}=-({\dot{\sigma }}_{\text{d}}(u_{l})+\epsilon )x_{j}\Theta \left(n^{\text{Ca}}-\sum_{k\in K}s_{k}^{\text{NMDA}}\right).$$

For clustering loss C we obtain


(21) $$C=\sum_{k}\text{Var}(s_{k}^{\text{NMDA}})$$

(22) $$\begin{array}{l} =\underset{k}{\sum }\sigma_{\text{d}}\left(u_{k}\right)\left(1-\sigma_{\text{d}}\left(u_{k}\right)\right) \end{array}$$

(23) $$\Rightarrow \frac{\partial C}{\partial w_{lj}}=x_{j}{\dot{\sigma }}_{\text{d}}(u_{l})(1-2\sigma_{\text{d}}(u_{l})).$$

We define a regularization term, which is added to the weight update, by


$$h_{j}(u_{l},w_{l})=s_{k}^{\text{NMDA}}\left[w_{lj}(\sum_{s}w_{ls}-1)+w_{lj}(1-x_{j}^{\text{apical}})\right],$$

where the first term normalizes the branch weights to an L1-norm of 1 and the second term reduces weights of inactive synapses, which are ignored by gradients of A and D. The weight update Δ*w*_*lj*_ is obtained as


$$\begin{array}{l} \Delta w_{lj}=\eta (w_{lj})\;[u^{\text{BP}}x_{j}f(u_{l})\Theta \left(n^{\text{Ca}}-\sum_{k\in K}s_{k}^{\text{NMDA}}\right)\\ \;+\lambda \;u^{\text{BP}}x_{j}g(u_{l})\left(2s_{k}^{\text{NMDA}}-1\right)\\ \;-\kappa \;\left(1-u^{\text{BP}}\right)x_{j}g(u_{l})\\ \;-\lambda_{\text{reg}}\;u^{\text{BP}}h_{j}(u_{l},w_{l})], \end{array}$$

with $f\left(u_{l}\right)={\dot{\sigma }}_{\text{d}}\left(u_{l}\right)+\epsilon$ and $g\left(u_{l}\right)={\dot{\sigma }}_{\text{d}}\left(u_{l}\right)$. We can show that the first term is a function of the calcium spike *S*^Ca^ as follows. Recall the definition


(25) $$s^{\text{Ca}}=\{\begin{array}{ll} 1, & \text{if }u^{\text{b}}\geq \theta^{\text{b}}\text{ and }u^{\text{a}}\geq n^{\text{Ca}}\\ 0 & \text{otherwise.} \end{array}$$

(26) $$=\Theta (u^{\text{b}}-\theta^{\text{b}})\Theta (-n^{\text{Ca}}+u^{\text{a}})$$

From *u*^BP^ = Θ(*u*^b^−θ^b^) we can re-write the product


(27) $$u^{\text{BP}}\Theta (n^{\text{Ca}}-u^{\text{a}})=\Theta (u^{\text{b}}-\theta^{\text{b}})\Theta (n^{\text{Ca}}-u^{\text{a}})$$

(28) $$=\Theta (u^{\text{b}}-\theta^{\text{b}})\left(1-\Theta (-n^{\text{Ca}}+u^{\text{a}})\right)$$

(29) $$=\left(\Theta (u^{\text{b}}-\theta^{\text{b}})-\underset{=S^{\text{Ca}}}{\underset{︸}{\Theta (u^{\text{b}}-\theta^{\text{b}})\Theta (-n^{\text{Ca}}+u^{\text{a}})}}\right)$$

(30) $$=\left(u^{\text{BP}}-S^{\text{Ca}}\right)$$

(31) $$=u^{\text{BP}}\left(1-S^{\text{Ca}}\right),$$

since Θ(*x*)·Θ(*x*) = Θ(*x*). The weight-dependent learning rate η(*w*_*lj*_) implements a soft-bound on weights by scaling down updates of weights close to the boundaries 0 and *w*_max_:


(32) $$\begin{array}{l} \eta \left(w_{lj}\right)=\eta^{\text{CAL}}w_{\text{max}}\left(\frac{w_{lj}^{2}\cdot {\left(w_{lj}-w_{\text{max}}\right)}^{2}}{{\left(\frac{w_{\text{max}}}{2}\right)}^{4}}+\frac{1}{40}\right), \end{array}$$

where apical dendritic learning rate η^CAL^ is a hyperparameter.

### 4.4 Details to unsupervised Hebbian K-winner-take-all learning

For the experiments in Sections 2.5 and 2.6, we applied a variant of the learning rule from Krotov and Hopfield (2019) with K-winner-take-all (k-WTA) from Section 4.1.2 to train our basal weights. This function slightly differs from the WTA activation function in the original paper, but we empirically observed that the feature representation learnt by k-WTA better suited this task. The update of basal weight *v*_*ji*_ from input neuron *i* to pyramidal neuron *j* for minibatch B was then defined by


(33) $$\Delta v_{ji}=\eta^{\text{basal}}\frac{1}{\underset{i,j}{\max}|\Delta {\bar{v}}_{ji}|}\Delta {\bar{v}}_{ji}$$

(34) $$\text{with }\Delta {\bar{v}}_{ji}=\sum_{x\in ℬ}q_{j}(x_{i}-u_{j}^{\text{b}}v_{ji})$$

where *q*_*j*_ was the basal activation $u_{j}^{\text{b}}$ of neuron *j* after applying k-WTA. As done in the code provided by Krotov and Hopfield (2019), we decayed learning rate η^basal^ linearly from an initial value of $\eta_{0}^{\text{basal}}=0.02$ to 0 through the 80 training epochs, where the learning rate for epoch *n* was given by $\eta_{n}^{\text{basal}}=\eta_{0}^{\text{basal}}\left(1-\frac{n}{80}\right)$.

### 4.5 Details to our extension to unsupervised Hebbian K-winner-take-all learning

We found that the feature representation after training with the k-WTA Krotov-rule (see previous section for details) resulted in multiple neurons tuning to the same feature value in the input. We hypothesize, that the reason for this behavior is that the weights of all *K* active neurons are updated toward the whole input vector **x**, regardless whether some neurons already encode features of the input. We applied principles from a Bayesian perspective on k-WTA learning (Legenstein et al., 2017) to extend the learning rule:


(35) $$\Delta {\bar{v}}_{ji}=\eta^{\text{basal}}\sum_{x\in ℬ}q_{j}(x_{i}-\sum_{l=1}^{N}q_{l}w_{li}),$$

where *N* is the number of neurons. Intuitively, neural weights are then only trained on the part of the input which is not yet explained by other neurons. We refer to this update rule as the Krotov+ rule.

### 4.6 Apical Excitation

The quantity *e*^a^, introduced in Section 2.2 can be calculated analytically as


$$\begin{array}{l} e^{\text{a}}=E_{s_{k}^{\text{NMDA}}\sim \sigma_{\text{d}}(u_{k})}[\Theta (u^{\text{a}}-n^{\text{Ca}})]\\ \;=1\cdot p(\sum_{k\in K}s_{k}^{\text{NMDA}}\geq n^{\text{Ca}})+0\cdot p(\sum_{k\in K}s_{k}^{\text{NMDA}}<n^{\text{Ca}})\\ \;=1-p(\sum_{k\in K}s_{k}^{\text{NMDA}}<n^{\text{Ca}})\\ \;=1-\sum_{r=0}^{n^{\text{Ca}}-1}p(\sum_{k\in K}s_{k}^{\text{NMDA}}=r). \end{array}$$

Note, that $p(\sum_{k\in K}s_{k}^{\text{NMDA}}=r)$ is the probability mass function of a Poisson-Binomial distribution, a generalization of the Binomial distribution to events with different probabilities. It is defined by


$$p(\sum_{k\in K}s_{k}^{\text{NMDA}}=r)=\sum_{A\in F_{r}}\prod_{i\in A}p_{i}\prod_{j\in A^{c}}\left(1-p_{j}\right),$$

where *F*_*r*_ is the set of all subsets of *r* integers that can be taken from {1, 2, …, *K*}. *A*^*c*^ is the complement of *A* such that *A*^*c*^ = {1, 2, …, *K*}\*A* (Wang, 1993). Probabilities *p*_*i*_ are thereby the NMDA spike probabilities σ_d_(*u*_*i*_). For *n*^Ca^ = 1, *e*^a^simplifies to


(36) $$e^{\text{a}}=1-\prod_{k\in K}\left(1-\sigma_{\text{d}}(u_{k})\right).$$

### 4.7 Pattern generation

For all tasks, all apical inputs were randomly pre-generated patterns by the following process: To generate the *n*-th of *N* population patterns $P_{n}\in {\left\{0,1\right\}}^{d}$ of dimension *d*, we randomly selected a sub-set of *a* dimensions which were set to 1, all others to 0. Then, the pairwise cosine-similarity between new pattern *P*_*n*_ and all *n*−1 other patterns was calculated. The pattern was rejected if this pairwise cosine-similarity exceeded a certain overlap threshold *o*_max_ to enhance separability of patterns. If sparse population activity is assumed with *a* < < *d*, the expected cosine similarity between two randomly generated patterns is very low, which is in accordance with sparse population coding in the brain (Rolls and Tovee, 1995).

### 4.8 Details to pattern association learning

In the pattern association learning experiment (Section 2.3), the model consisted only of apical dendrites, without considering basal dendrites. We split this experiment into four parts, corresponding to Figures 3A, C–E.

**Figure 8:**
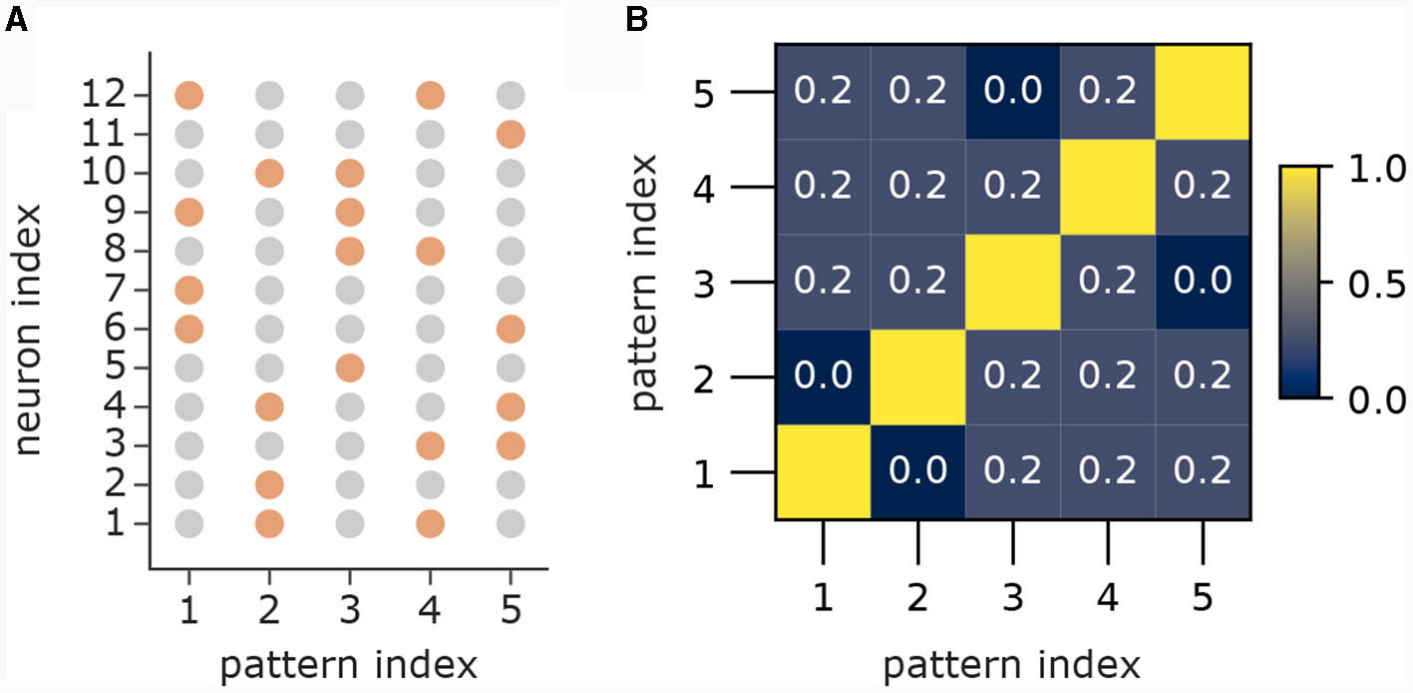
Patterns from first part of pattern learning experiment. **(A)** The population patterns used for the pattern learning experiment from Figure 3A. Orange circles denote active inputs ($X_{i}^{\text{apical}}=1$), gray circles denote inactive inputs ($X_{i}^{\text{apical}}=0$). **(B)** Pairwise cosine similarity between patterns.

In the first part (Figure 3A) we showed 5 patterns *P*_1_, …, *P*_5_ sequentially to the apical dendrites with 5 branches. The apical input vector *x*^apical^(*t*) at time step *t* was thereby set to *x*^apical^(*t*) = *P*_1_ for 0 ≤ *t* < 80, then to *x*^apical^(*t*) = *P*_2_ for 80 ≤ *t* < 160, followed by patterns 3, 4 and 5 until time step 399. To improve visibility in the plot in Figure 3A), we added 20 time steps with *x*^apical^(*t*) = **0**, followed by a re-presentation of each pattern for 10 time steps with 3 time steps of **0** in between to again improve visibility. We set back-propagating activity *u*^BP^(*t*) = 1 at each time *t*, so that only the association terms of the learning rule were engaged. Patterns 1 to 5 were generated by the generation process described in Section 4.7 with dimensionality *d* = 12 with *a* = 4 active dimensions and overlap threshold of *o*_max_ = 0.4. The patterns for this experiment alongside their pairwise cosine similarity are shown in Figure 8.

For the second part, we used 21 branches and 21 patterns and assigned a mean back-propagating activity level of $p_{p}^{\text{BP}}$ in the interval [0, 1] to each pattern *P*_*p*_. The assigned levels for each pattern were distributed with equal spacing between $p_{1}^{\text{BP}}=0$ and $p_{21}^{\text{BP}}=1$ (see also Figure 3Ci). The patterns were randomly presented for a total of 8400 time steps, where at each time step *t* the apical input was *x*^apical^(*t*) = *P*_*l*_ where *l* was uniformly drawn from 1, …, 21. We further sampled a binary ${ū}^{\text{BP}}\left(t\right)\sim B\left(p_{l}^{\text{BP}}\right)$ from a Bernoulli distribution with mean $p_{l}^{\text{BP}}$, which was then given as back-propagating activity *u*^BP^(*t*). We generated patterns of dimension 400 with 40 active neurons as described in Section 4.7. Weight updates were computed at each time step according to Equation 13. The dendritic learning rate was set to η = 0.08. For high input dimensionality we found it beneficial to introduce sparsity in the weight initialization, which increases the variance of branch outputs in response to patterns, reducing the time required for a branch to win the competition. Hence we set 40% of the synaptic efficacies on each dendritic branch to 0, whereas all other synapses were initialized from a Gaussian distribution with mean 0.4*w*_max_ and standard deviation of 0.1*w*_max_ and clipped in the interval [0, *w*_max_], where $w_{\text{max}}=\frac{1}{40}$.

In the third part of the experiment, we modeled 10 apical dendritic branches, whereas the patterns were the same as in the previous part of the experiment. $p_{p}^{\text{BP}}$ in this experiment were chosen in the interval [0.5, 1] and (as in the experiment before) assigned equally spaced to patterns *P*_1_ to *P*_21_. Total number of time steps, pattern presentation order and initialization were chosen as in part two of the experiment described in the previous paragraph.

In the fourth experiment, we set the pattern size and number of active dimensions in the patterns to 600 and 90 respectively, as well as enforced 90% of randomly selected connections to each dendritic branch to constant 0. This way, we modeled sparse connectivity from apical input neurons (represented by the apical input *x*^apical^) to the dendritic branches. We generated 40 different patterns, assigned $u_{p}^{\text{BP}}=1$ to half of the patterns (randomly chosen), and $u_{p}^{\text{BP}}=0$ to the other half. We ran the experiment with a Ca^2+^ spike threshold of *n*^Ca^ = 2 (for *n*^Ca^ = 3 see Supplementary material Section S3). Patterns were presented sequentially as in the first part of the experiment. After all patterns were learnt, we finally measured the apical excitation *e*^a^ for 1000 presentations of each pattern (with CAL disabled).

### 4.9 Details to population-level coincidence detection via CAL

In the population coincidence experiment from Section 2.4 and Figure 4, we randomly generated 10 patterns *P*_1_, …, *P*_10_, as described in Section 4.7, of dimension 400 with 40 active dimensions per pattern with a maximum overlap of 30%. These patterns served as context input to apical dendrites. For each basal stimulation distribution S_*p*_, we randomly selected a specific sub-set of 20 neurons from the population of 60 neurons. This subset of neurons was strongly stimulated (*u*_*b*_~N(0.7, 0.2)) whenever we presented the corresponding context pattern *P*_*p*_. All other neurons received weak stimulation (*u*_*b*_~N(0.3, 0.2)). At each time step, we randomly presented a context *P*_*p*_ together with basal stimulation according to basal stimulation distribution S_*p*_, for 2000 time steps in total. The reported values in Figures 4C–E are averages over 10 runs with different apical dendritic branch initializations, but the same context patterns and the same sub-sets of highly stimulated neurons for each context.

### 4.10 CDFA dataset

One sample in the CDFA task (used for experiments in Sections 2.5 and 2.6) is a triplet (**f**, **c**, ŷ) with feature vector **f** ∈ {0, 1}^*nm*^, context **c** ∈ {0, 1}^60^ and target ŷ ∈ {0, 1}. Feature vector **f** is thereby a concatenation of *n* = 6 individual feature values **f** = concat(**f**_1_, …, **f**_*n*_) of length *m* = 100, where each feature value **f**_*l*_ is instantiated from a set F_*l*_ of 10 different values. Each of the values is a set-wide unique, pre-generated pattern, whereas patterns can re-occur in multiple sets. The random patterns for each set F_*l*_ are pre-generated according to the procedure described in Section 4.7 with pattern dimension 100 and 20 active neurons per pattern. Despite the above mentioned constraint of uniqueness of feature value patterns within a set F_*l*_, the overlap between patterns was not limited. The expected overlap between two patterns can be computed as expectation of the corresponding hypergeometric distribution and is ≈20% for the above described pattern configuration. Class vector **c** is drawn from a pre-generated set C = {**c**_1_, **c**_2_, …, **c**_*K*_} of *K* different random patterns, each representing the identity of a class. The patterns are again sampled according to the procedure described in Section 4.7, with pattern dimension 60 of which 9 neurons where active. The maximum overlap of active neurons between patterns was limited to 40%. We assign a specific subset of 3 randomly drawn feature values (with a maximum of 1 from each feature set F_*l*_) to each class *k*, which we call class definition D_*k*_. The network does not have access to this class definition, since this definition gives the solution of which feature values are related to which class, and instead should be learned by the network from the data. From the class definition sets D_*k*_, the feature value sets F_*l*_ and the set of class identities C we can then generate a data set for training and testing the network. For generating this data set, we apply the procedure described in the following. First, we randomly choose a class *k*. Then we generate a feature vector **f** matching to this class. This is done by setting the appropriate feature values in **f** from the class definition D_*k*_. The rest of the features (which we call don't care features for this class) are then randomly set to values from the corresponding feature sets F. The triplet (**f**, **c**_*k*_, ŷ = 1) then represents a new positive data sample, where **c**_*k*_ is the *k*-th pre-generated pattern from C for class *k*. To ensure balance between positive (ŷ = 1) and negative (ŷ = 0) samples, we then choose a random class $\bar{k}$, ensure that the class does not match to feature vector **f** (which can happen by chance) and add this new triplet $\left(f,c_{\bar{k}},\hat{y}=0\right)$ as negative data sample to the data set.

The data set for unsupervised training of basal weights was generated using a slightly different procedure: We generated a separate data set consisting of 1, 000 feature vectors with each feature value *l* randomly drawn from the set of values F_*l*_. No contexts or targets were added and the inputs were not constrained to resemble any classes. The point of this procedure was to establish an unbiased feature representation in the basal weights.

### 4.11 Details to context-feature association

The network model for solving this task (Section 2.5) consisted of 600 “bottom-up” input neurons, which were fully connected to the basal compartments of a hidden layer of 60 pyramidal neurons, followed by a single output neuron, receiving input from all pyramidal neurons.

The pyramidal cells received as basal input the visual features in the following way. The value of each feature *i* (e.g., “shape”, “color”, etc.) was encoded as a binary vector $f_{i}\in {\left\{0,1\right\}}^{m}$ of length *m* = 100 which took on a different binary pattern for each distinct value of the feature (randomly chosen). For example, the “color” feature vector has different patterns for “red”, “blue”, “yellow”, etc. The total feature vector **f** ∈ {0, 1}^*nm*^ was then the concatenation of the *n* individual feature values **f** = concat(**f**_1_, …, **f**_*n*_). In our simulations, we used *n* = 6 features. The apical input to the neurons consisted of the context object class, each encoded as a class vector **c** ∈ {0, 1}^60^ that was a randomly generated unique pattern of exactly 9 non-zero elements.

Each pyramidal neuron was equipped with 10 apical branches, where each branch in each neuron received the same input *x*^apical^ ∈ {0, 1}^60^ from 60 “top-down” input neurons. Hence, the number of synapses on each branch matched the dimension of *x*^apical^, which was 60. We trained the network in two stages: during the first stage, we showed a set of random feature value compositions (see also Section 4.10) to the basal weights of the pyramidal layer using the bottom-up input neurons. The 1, 000 samples were presented for 80 iterations, basal weight updates were calculated in minibatches of 16 using either the k-WTA-variant of the Krotov learning rule described in Section 4.4 or the Krotov+ learning rule described in Section 4.5. This training stage was class agnostic, hence the apical dendrites did not receive any context input. After this first stage, the basal weights were held fixed, and we presented training data consisting of in total 20, 000 samples of 100 different object classes, with context inputs, as described in Section 4.10. Feature vectors **f** were presented using the bottom-up input neurons and the context vectors **c** via the top-down input neurons. Weight updates to the apical dendritic synapses, according to the CAL rule (as defined in Section 4.3), were performed in minibatches of 64 with a dendritic learning rate of η = 0.06. In addition, we trained the threshold θ of the output neuron with a cross-entropy loss on the target using the ADAM optimizer. The Heaviside step function was thereby replaced by the logistic sigmoid to ensure non-zero gradients. K-winner-take-all as described in Section 4.1.2 was applied to the basal outputs with the same *K* used during training of basal weights. We measured test accuracy on 5, 000 samples held-out for training. For this performance evaluation, we replaced the binary calcium spike *S*^Ca^ with its expectation to compute the output rate of neurons, i.e., we used *S*^Ca^ = Θ(*u*^b^−θ^b^)*e*^a^. This ensured deterministic network behvior during test time.

### 4.12 Details to continual learning

In the continual learning scenario (from Section 2.6) of the CDFA task, we performed training in a three-stage process, where the first two stages were equivalent to the training procedure described in Section 4.11. In the third stage, the continual learning stage, we showed additional mini data sets, each of which depicting an individual new and unobserved task, sequentially without re-presentation of data from previous data sets. Note, that training on new data without re-training on previous data is the main difference between the two related concepts of multi-task learning and continual learning.

For the continual learning scenario of the CDFA task set (see Figure 7), a full data set with 11, 520 samples, and a data set with 1, 000 samples were generated as described in Section 4.10. The 1, 000 samples were only used for the first training stage, unsupervised training of basal weights, and consisted only of random feature value compositions (see also Section 4.11). A further split of the full data set into multiple sub-sets, one sub-set per task, was required. We performed this split by first grouping the data samples by context into groups G_1_, …, G_48_, where in each group G_*k*_ only data samples with common context vector **c**_*k*_ occurred. Then we merged the samples from the first 40 groups into an initial data set. This initial data set was used in the second stage of training, where basal weights were already frozen, and training of apical synapses with CAL, together with supervised training of the readout threshold θ were enabled and applied for 120 episodes with mini-batch size 64. The remaining 8 groups G_41_, …, G_48_ then provided the data sets for each of the 8 mini-tasks respectively, which were learned by the network incrementally in the third and last stage of training, where the readout threshold θ was fixed and only CAL was active. 20% of the 11, 520 data samples were held-out from training, split into subsets as described above, and used for testing.

### 4.13 Hyperparameters

We report hyper-parameters for the pattern learning experiment (Section 2.3 and Figure 3) in Table 4, for the coincidence detection experiment (Section 2.4 and Figure 4) in Table 5, hyper-parameters specific to the CDFA task (Section 2.5 and Figures 5 and 6) in Table 6 and to the continual variant of the CDFA task (Section 2.6 and Figure 7) in Table 7.

**Table 4:** Parameters of the pattern learning scenarios from different panels of Figure 3.

**Parameter**	**Experiment (panel)**			
	**1 (A)**	**2 (C)**	**3 (D)**	**4 (E)**
Sparse connections	No	No	No	Yes
λ	0.33	0.33	0.33	0.33
κ	0.3	-	0.3	0.3
Apical input pattern size	12	400	400	600
Active components in apical input patterns	4	40	40	90
λ_reg_	4	40	40	0.09
Ca^2+^ spike threshold *n*^Ca^	1	1	1	2
*w*_max_	1/4	1/40	1/40	0.123
Number of apical input patterns	5	21	21	40
Number of apical dendritic branches	5	21	12	12
μ^init^	0.4*w*_max_	0.4*w*_max_	0.4*w*_max_	0.6*w*_max_
σ^init^	0.1*w*_max_	0.1*w*_max_	0.1*w*_max_	0.2*w*_max_
η^CAL^	0.04	0.04	0.04	0.06
ap. init. sparsity	0%	40%	40%	40%
ap. perm. sparsity	0%	0%	0%	90%

Apical dendritic weights are initialized from a normal distribution with mean μ^init^ and variance σ^init^. η^CAL^: learning rate for CAL rule (see Equation 33). ap. init. sparsity.: Percentage of weights per apical branch set to 0 at initialization. ap. perm. sparsity.: Percentage of apical weights forced to 0 during the entire experiment.

**Table 5:** Parameters of population-level coincidence detection experiment.

**Parameter**	**Value**
λ	0.33
κ	0.3
α	5
apical input pattern size	400
active components in apical input patterns	40
λ_reg_	40
*w*_max_	1/40
ap. weight init.	N(0.4*w*_max_, 0.1*w*_max_)
apical dendritic branches per neuron	10
η^CAL^	0.08
number of different contexts	10
training epochs	2000
neuron population size	60
strongly stimulated neurons per context	20
high stimulation	N(0.7, 0.2)
low stimulation	N(0.3, 0.2)
θ^b^	0.5
ap. init. sparsity	40%

N(μ, σ^2^) denotes a normal distribution with mean μ and variance σ^2^. ap. weight init.: initialization of apical weights. η^CAL^: learning rate for CAL rule (see Equation 33). Training epochs: Number of randomly drawn pattern presentations, 2000 epochs with 10 contexts means that each context pattern was shown on average 200 times in total.

**Table 6:** Parameters of the context-dependent feature association experiment.

**Parameter**	**Value**
λ	0.33
κ	0.3
λ_reg_	18
α	10
Apical input pattern size	60
Active components in apical input patterns	9
*w*_max_	1/9
ap. weight init.	N(0.016, 0.03)
bas. weight initialization	N(0, 1)
Apical dendritic branches per neuron	10
η^CAL^	0.08
Neuron population size	60
ap. init. sparsity	40%
θ initialization	20
*K* for K-WTA	6
Classes	[10, 450]
Features	6
Values per feature	10
Neurons per feature	100
Active neurons per feature	20
Features defining a class	3
Training samples	250· # of classes
Episodes for unsupervised training of basal weights	80
Unsupervised basal training batch size	16
Training episodes	100
Batch size	64

N(μ, σ^2^) denotes a normal distribution with mean μ and variance σ^2^. ap. weight init.: initialization of apical weights. η^CAL^: learning rate for CAL rule (see Equation 33).

**Table 7:** Specific parameters of the continual context-dependent feature association experiment.

**Parameter**	**Value**
Total number of classes	48
Thereof used for pretraining	40
Training samples	240· # of classes
Training episodes for pretraining (joint training of first 40 classes)	120
Training episodes for tasks 1 to 8 (continual)	60 per task

All other parameters were chosen as in the CDFA multi-task experiment, see Table 6. “Training episodes for pretraining” denotes the number of episodes in which the first 40 classes were jointly trained to learn a threshold for the readout neuron (see also main text).

## Data availability statement

The raw data supporting the conclusions of this article will be made available by the authors, without undue reservation.

## Author contributions

MB: Conceptualization, Investigation, Methodology, Software, Writing – original draft, Writing – review & editing. RL: Conceptualization, Funding acquisition, Investigation, Supervision, Validation, Writing – review & editing, Methodology, Writing – original draft.
