# Supplementary material for: Context association in pyramidal neurons through local synaptic plasticity in apical dendrites
Source: Front Neurosci. 2024 Jan 31;17:1276706. doi: 10.3389/fnins.2023.1276706 (PMC10864492; doi:10.3389/fnins.2023.1276706)
Supplement: Supplementary file 1 [file Data_Sheet_1.pdf]

Context association in pyramidal neurons through local synaptic plasticity in apical dendrites - Supplementary Material

Maximilian Baronig and Robert Legenstein

Institute of Theoretical Computer Science, Graz University of Technology, Graz, Austria

Correspondence*:
Robert Legenstein
robert.legenstein@igi.tugraz.at

S1 PHASE PLANE ANALYSIS OF THE CAL RULE WITHOUT CLUSTERING OBJECTIVE

Figure S1 shows the phase plane analysis as in Figure 2 of the main text for $u^{\mathrm{BP}} = 1$ with disabled clustering loss. The clustering loss was disabled by setting $\lambda = 0$. One can see that the dynamics point to a configuration where both mean weights reach their maximum. Thus, there is no competition between the two branches (compare to Figure 2Cii).

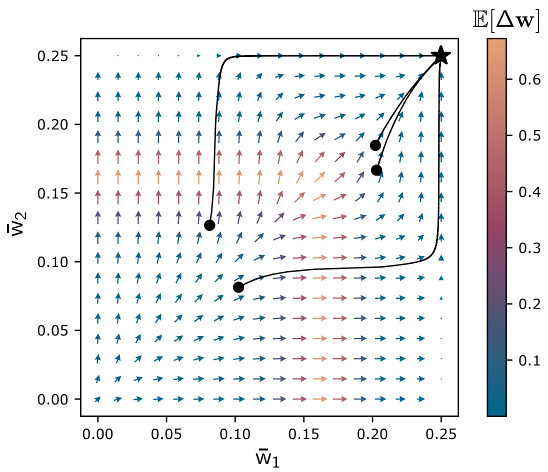

Figure S1. Phase plane analysis from Figure 2 with $\lambda = 0$ and $u^{\mathrm{BP}} = 1$.

S2 PATTERN LEARNING WITH SPARSE CONNECTIONS

Figure S2 shows the pattern learning experiment from Figure 3A where 90% of apical input weights were constrained to 0. The apical pattern size was 600 where 90 elements were active for each pattern and presented the patterns sequentially (as in Figure 3A), such that each pattern was presented for 80 iterations

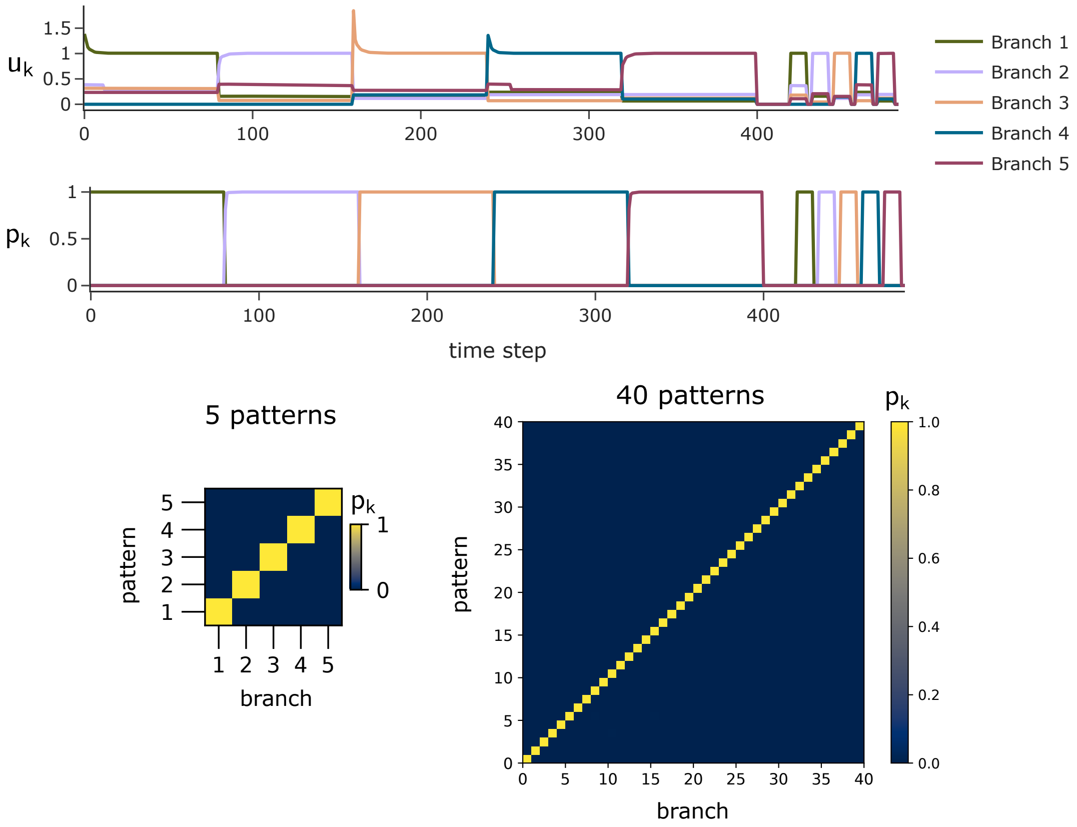

Figure S2. Pattern learning with sparse connections. Same as panels A and Biii of Figure 3 in the main text.

in a row. Figure S2 shows results for 5 patterns and branches as well as for 40 patterns and branches. Notably, even in the case of sequentially learning 100 patterns with 100 branches, each pattern depolarized one single unique branch (result not shown).

S3 PATTERN LEARNING WITH HIGHER CALCIUM SPIKE THRESHOLD

We considered the configurations $n^{\mathrm{Ca}} \in \{2, 3\}$ for the sequential pattern learning experiment with sparse connections (see also Section S2). We generated 40 context patterns with a pattern size of 600, of which 90 components were active in each pattern. We randomly assign a-priori a static $u^{\mathrm{BP}} = 1.0$ to 20 of the patterns, and a $u^{\mathrm{BP}} = 0.0$ to the other 20. Patterns were presented sequentially one at a time, along with the pre-assigned level of u^{BP}. Figures S3 and S4 show the results for $n^{\mathrm{Ca}} = 2$ and $n^{\mathrm{Ca}} = 3$ respectively. Parameters were the same as in the experiment of Figure 3E in the main text, with the following exceptions for $n^{\mathrm{Ca}} = 3$: $\lambda_{\mathrm{reg}} = 0.009$, $w_{\mathrm{max}} = 0.152$ and weight initialization for apical dendritic weights was sampled from $\mathcal{N}(0.4 w_{\mathrm{max}}, 0.1 w_{\mathrm{max}})$.

S4 ANALYSIS OF THE NETWORK CAPACITY IN THE CDFA TASK

In Figure 6C in the main text, a decrease in performance can be observed for an increasing number of object classes. If we assume an orthogonal representation of feature values, then each class requires 3 apical dendritic branches on different neurons (since each class is uniquely defined by 3 feature values). If we assume a uniform distribution of feature values across classes (which is valid for our task), the expected number of branches b is $\mathbb{E}[b] = \frac{3}{60} c$ with number of classes c. Solving for c, we obtain $c = \frac{60}{3} b = \frac{600}{3} = 200$,

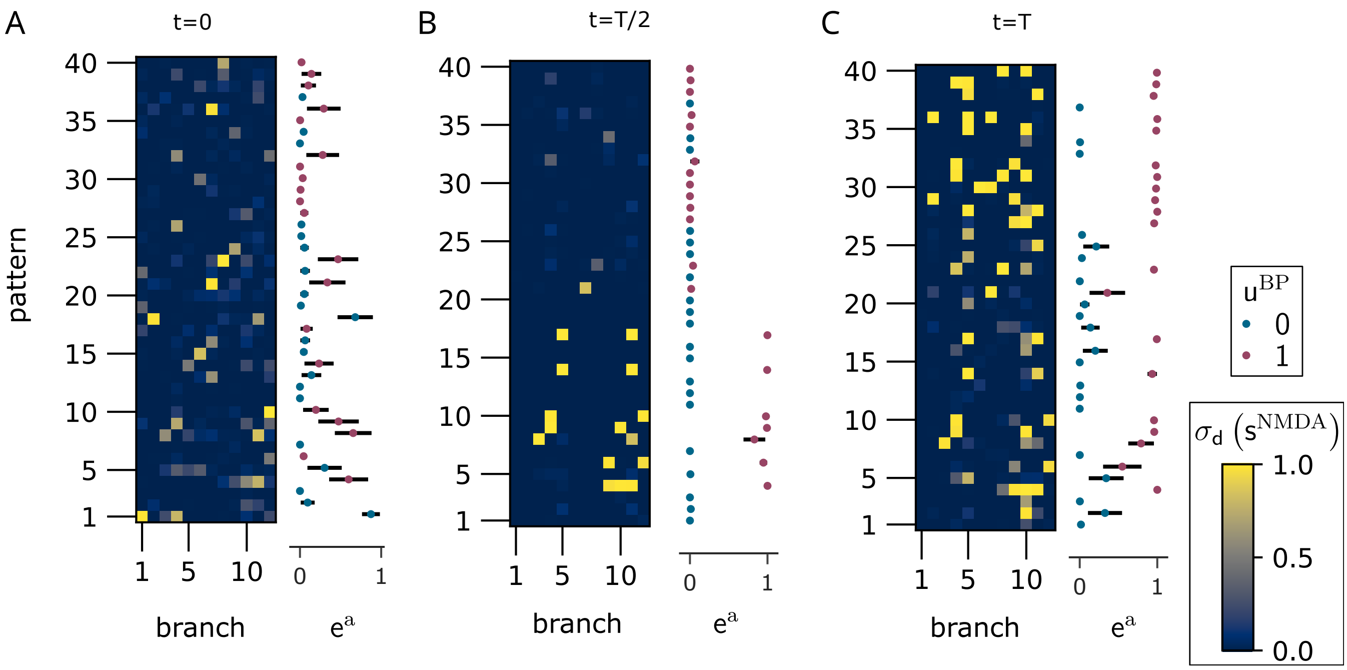

Figure S3. Sequential pattern learning experiment with $n^{Ca} = 2$. **(A)** Tuning matrix after initialization. Color of dots indicates the u^{BP} that was a-priori assigned to each pattern. **(B)** Tuning matrix after half of the total training time steps T, where 20 of the 40 patterns were learnt. Patterns 1 to 20 were presented together with the u^{BP} indicated by the dot color. Of the learnt patterns, the ones with $u^{BP} = 1$ result in higher apical excitation e^a than patterns with $u^{BP} = 0$. All learnt patterns result in activation of 2 dendritic branches, which is a consequence of setting Ca^{2+}spike threshold $n^{Ca} = 2$. **(C)** Tuning matrix after all patterns have been learnt.

since we use 60 neurons with 10 apical dendritic branches each. The drop in performance with more than 200 classes is therefore due to exhaustion of dendritic branch capacity.

S5 CONTINUAL LEARNING WITH KROTOV+

In addition to the continual learning plot in the main text, we show the performance of the network in the continual learning task if the Krotov+ rule is used for unsupervised learning of basal weights instead of the Krotov rule, see Figure S5.

S6 COMPARISON BETWEEN DENDRITIC LOGISTIC REGRESSION AND THE CAL RULE

Dendritic Logistic Regression (DLR) has been proposed as a local learning rule that predicts basal activity from apical input (Rao et al., 2022). Since this objective is close to the objective of the CAL rule, we wondered how DLR would perform in the CDFA task set. We used the same network setup as for the CAL rule where basal weights were trained with the Krotov rule. On the basic CDFA task, the error with the DLR rule was $20.27 \pm 2.34\%$ (CAL: $6.89 \pm 1.32\%$). This shows that, while DLR can associate contextual patterns, its performance is substantially below the performance of the CAL rule. In the continual learning setup from Section 2.6, the error of the DLR rule was $30.96 \pm 2.45\%$ (CAL: $6.81\% \pm 10.54\%$). This result indicates that clustering of synaptic patterns on apical branches is benefits continual learning. Figure S6

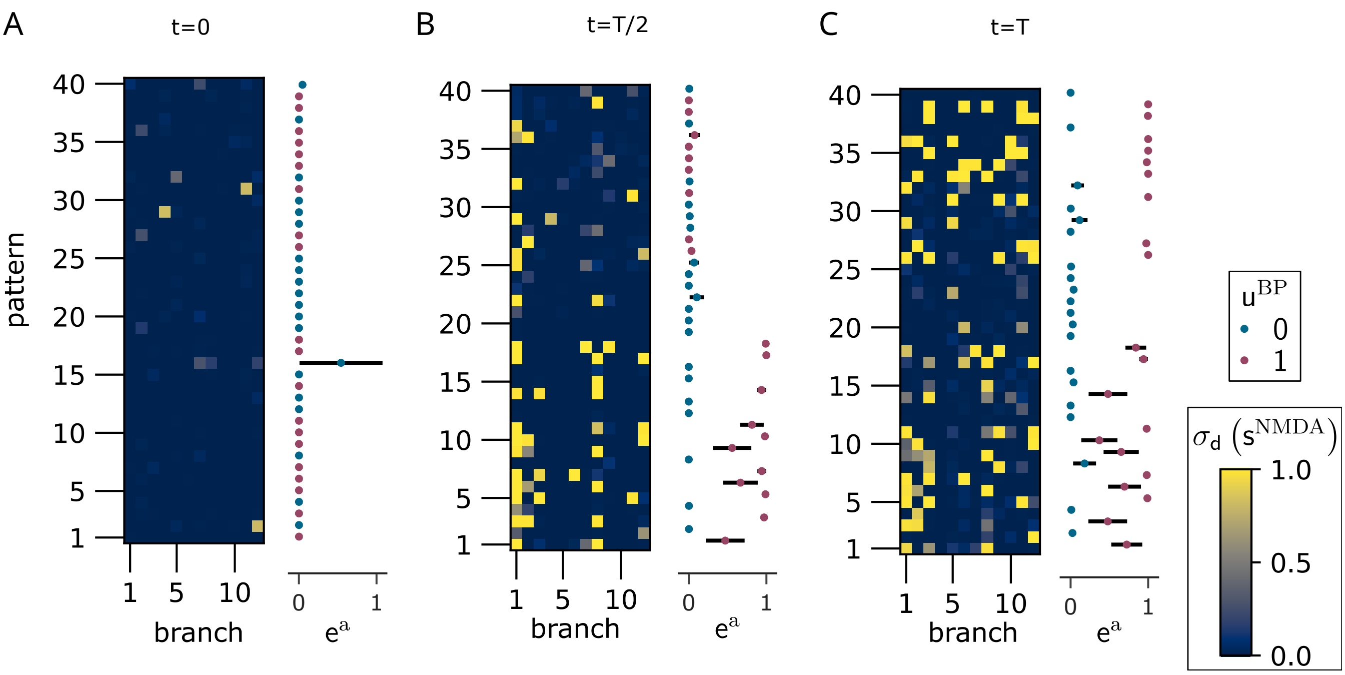

Figure S4. Same as previous Figure, but with $n^{\mathrm{Ca}} = 3$.

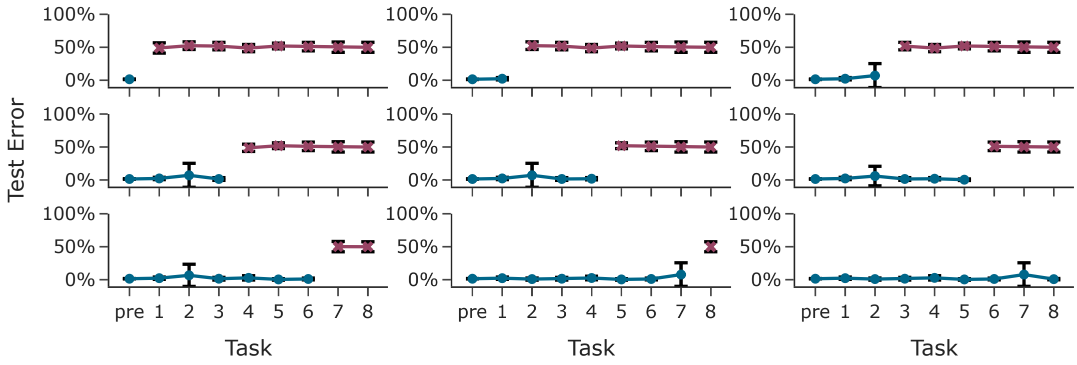

Figure S5. Continual learning with Krotov+ rule. Same as Figure 7A in main text.

shows detailed results of the continual learning setup with DLR. The DLR-specific hyper-parameters (see Rao et al. (2022)) were $\alpha = 8$, $\theta = 8$, $u_0 = 0$, $\beta = 1$ and a learning rate for apical dendritic weights of 0.1.

REFERENCES

Rao, A., Legenstein, R., Subramoney, A., and Maass, W. (2022). Self-supervised learning of probabilistic prediction through synaptic plasticity in apical dendrites: A normative model. *bioRxiv* doi:10.1101/2021.03.04.433822

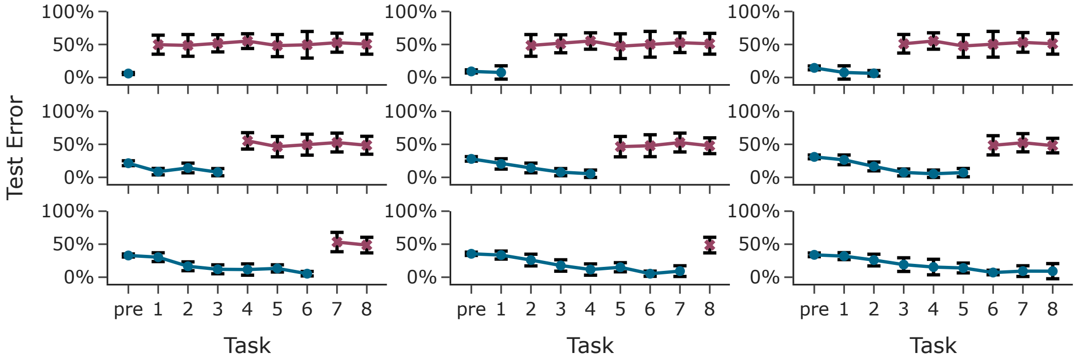

Figure S6. Continual learning with Dendritic Logistic Regression. Same as Figure 7A in main text, but with the DLR rule.
